# Pharmaceutical and Trace Metal Interaction within the Water–Soil–Plant Continuum: Implications for Human and Soil Health

**DOI:** 10.3390/toxics12070457

**Published:** 2024-06-25

**Authors:** Lesly Ayala Cabana, Ana de Santiago-Martín, Raffaella Meffe, Isabel López-Heras, Irene de Bustamante

**Affiliations:** 1 IMDEA Water Institute, Alcalá de Henares, 28805 Madrid, Spain; ana.desantiago@imdea.org (A.d.S.-M.); raffaella.meffe@imdea.org (R.M.); isabel.lopez@imdea.org (I.L.-H.); irene.bustamante@imdea.org (I.d.B.); 2 Department of Geology, Geography and Environment, University of Alcalá, Alcalá de Henares, 28802 Madrid, Spain

**Keywords:** pharmaceuticals, transformation products, trace metals, interaction, drug–metal complexes, interstitial water, soil, lettuce, human health risk assessment, soil health

## Abstract

Unplanned water reuse for crop irrigation may pose a global health risk due to the entry of contaminants into the food chain, undesirable effects on crop quality, and impact on soil health. In this study, we evaluate the impact derived from the co-occurrence of pharmaceuticals (Phs), trace metals (TMs), and one metalloid within the water–soil–plant continuum through bioassay experiments with *Lactuca sativa* L. Results indicate that the co-occurrence of Phs and TMs has synergistic or antagonistic effects, depending on target contaminants and environmental compartments. Complex formations between drugs and TMs may be responsible for enhanced sorption onto the soil of several Phs and TMs. Concerning plant uptake, the co-occurrence of Phs and TMs exerts antagonistic and synergistic effects on carbamazepine and diazepam, respectively. With the exception of Cd, drugs exert an antagonistic effect on TMs, negatively affecting their uptake and translocation. Drug contents in lettuce edible parts do not pose any threat to human health, but Cd levels exceed the maximum limits set for leafy vegetable foodstuffs. Under Ph-TM conditions, lettuce biomass decreases, and a nutrient imbalance is observed. Soil enzyme activity is stimulated under Ph-TM conditions (β-galactosidase) and Ph and Ph-TM conditions (urease and arylsulfatase), or it is not affected (phosphatase).

## 1. Introduction

Water resources downstream of urban areas are very often impacted by the discharge of wastewater treatment plant (WWTP) effluents that discharge every day a wide range of chemicals into the environment [1,2]. Pharmaceuticals (Phs) and trace metals (TMs), among others, constitute a significant proportion of these effluents [3] due to their persistence despite the treatments in conventional WWTPs. The co-occurrence of Phs and TMs in environmental matrices can facilitate the formation of Ph-TM complexes, which are typically more persistent and toxic than the individual compounds [4]. This behaviour is of particular concern for antibiotics, as the formation of these complexes may enhance the persistence and proliferation of antibiotic-resistant bacteria [5]. When water resources substantially impacted by the discharge of WWTP effluents are used for crop irrigation (i.e., unplanned water reuse) [6], serious implications for human and soil health may arise.

Due to the increasing need to treat ageing-related and chronic diseases, worldwide Ph consumption is growing. Between 2000 and 2015 in the OECD countries, the consumption of cholesterol-lowering Phs has increased nearly fourfold, antidepressant use has doubled, and the use of antihypertensives and antidiabetics has nearly doubled [7]. Despite the extensive administration of approximately 2000 drugs worldwide, most have not been evaluated for their environmental occurrence, fate, or potential impacts on water quality, ecosystems, and human health [8]. Most chronic diseases are probably the result of a combination of genetics and environmental factors [9]. The ingestion of foods with low amounts of contaminants, such as drugs and TMs, represents a route of chronic exposure, with unpredictable consequences. Indeed, food consumption is the primary route of TM exposure in humans (>90% of the daily intake of total TMs). It has been widely demonstrated that chronic exposure to TMs results in various harmful effects, including reproductive toxicity, neurotoxicity, and cancer, among many others [10]. For example, Cd accumulation during the foetal stage is linked to a higher risk of developing metabolic diseases and endocrine disorders in adulthood (e.g., obesity, diabetes, etc.) [11]. Nonetheless, the adverse effects of many contaminants, as well as the complex interactions between them, are still unknown. Consequently, the true impact of contamination on the global disease burden is likely underestimated [9]. The characterization of contaminant exposures and possible adverse effects (namely exposome) of contaminants in multicomponent mixtures, from conception onward over a complete lifetime, is essential. This is especially true for children, for whom even low contaminant levels in utero and in early infancy can disturb their development and lead to disease in childhood or even later in life [9,12,13]. In this sense, Ph-TM complexes can be considered as contaminants of emerging concern, and the evaluation of their exposure should be addressed, considering their joint presence in the environment, which represents a great challenge [5,14,15].

The co-occurrence of Phs and TMs in surface waters impacted by WWTP effluents used for crop irrigation may produce a synergistic or antagonistic effect in their behaviour in the water–soil–plant continuum [16,17]. In soils, Phs can be combined with TMs by cationic bridge or surface complexes with different sorption affinities onto colloids [18,19], affecting their mobility in the soil–water interface and, hence, their bioavailability towards plants and soil microorganisms. Their behaviour at the soil–water interface can be very different as a function of the different affinities of Phs and TMs for soil colloids. Thus, Zhao et al. [20] observed that tetracyclines showed no effect on Pb adsorption, while Pb enhanced the tetracycline adsorption, especially under alkaline conditions, attributed to the higher binding affinity of Pb to soils. Given the importance of reactivity with soil components in sorption–desorption processes, studying the Ph-TM interaction in the soil–water interface is pivotal to obtain robust conclusions. Most of the studies focused on Ph-TM interaction in environmental matrices are performed with antibiotics of different classes, mainly sulphonamides [18], fluoroquinolones [4], and tetracyclines [20,21,22,23]. This is probably due to the usual coexistence of such antibiotics and TMs in soils through fertiliser and manure application. At a clinical and pharmacological level, studies about the effects on humans of complexes have been widely evaluated, either by unwanted interactions that increase or decrease the action of a given drug (e.g., interaction of atenolol and As, [24]), or by deliberately enhancing the therapeutic action of a drug (e.g., clarithromycin and Cu, [25]). If these effects exist at a clinical level, it is likely that they also exist to a greater or lesser extent after ingesting these contaminants through water or food; however, the literature on this topic is rather scarce. Concomitantly, there is an important gap in the knowledge about the water–soil–plant continuum about the effect of the coexistence of TMs and drugs other than antibiotics, which are usually present in higher concentrations in surface water affected by WWTP effluents. Most of available literature deals with drug–metal complexes from the clinical, industrial, and analytical fields: anti-inflammatories [26,27], antidiabetics [28], antiepileptics [29], anxiolytics [30,31], antidepressants [32], antihypertensives [33,34], and also antibiotics [25,35,36], among others. Also, the Ph-TM interaction may slow down Ph biodegradation processes, impacting crop development, Ph and TM uptake, and microbial activity to different degrees. In this sense, studies that evaluate the biodegradation rate of Phs in the presence or absence of TMs and how this affects their uptake by plants and the impact on soil health are essential. A healthy living soil is crucial for food security and nutrition [37]. Any alteration, whether positive or negative, on soil enzymes due to contamination could alter nutrient cycling, C sequestration, fertility, and, globally, soil health [38]. In this sense, enzyme activities are sensitive indicators of soil health alteration caused by Phs, TMs, and their co-occurrence [38,39,40], and they may provide useful information about the responses of soil to the disturbance caused by contamination. For example, Yang et al. [38] reported that the combined application of Cu and enrofloxacin produced greater toxicity in soil enzyme activities than their individual applications. 

The use of water resources impacted by WWTP effluents to irrigate crops may pose a global health risk due to the entry of contaminants and their transformation products (TPs) into the food chain, undesirable effects on crop quality, and an impact on soil health. Therefore, the general objective of this study is to evaluate the impact derived from the co-occurrence of Phs and TMs in the context of unplanned water reuse for crop irrigation within the water–soil–plant continuum. Specifically, this research aims to (i) investigate Ph and TM behaviour in the soil–water interface over time; (ii) study Ph, TP, and TM accumulation in the soil; (iii) assess Ph, TP, and TM plant uptake and translocation; (iv) evaluate the risk to human health derived from target crop consumption; (v) identify possible effects on crop quality; and (vi) assess the impact on soil health, considering hydrolase enzyme activity. To this end, a bioassay experiment with *Lactuca sativa* L. was conducted. The species was selected because of its outstanding potential for contaminant uptake, its wide consumption in the Mediterranean area, and its method of consumption (i.e., in the Mediterranean diet, it is usually consumed raw). A group of 22 Phs belonging to different therapeutic classes, 10 TPs, six TMs, and one metalloid were selected according to previous studies carried out in an unplanned water reuse scenario in the Mediterranean area [3,41].

## 2. Material and Methods

### 2.1. Chemicals and Reagents

A total of 22 Phs of different therapeutic classes and 6 TMs and 1 metalloid (hereinafter jointly referred to as TMs) were chosen. Selection was done based on a previous study carried out in the Manzanares River, which receives effluents from the largest WWTPs of Madrid (Madrid, Spain) (de Santiago-Martín et al., 2020). Indeed, WWTP effluent discharges constitute up to 83% of the Manzanares River flow, a common condition in the Mediterranean area. The 22 Phs selected were acetaminophen (ACE), atenolol (ATE), carbamazepine (CBZ), citalopram (CIT), clarithromycin (CLA), codeine (COD), diazepam (DIA), diclofenac (DIC), enalapril (ENA), flecainide (FLE), gemfibrozil (GEM), ibuprofen (IBU), lincomycin (LIN), lorazepam (LOR), metamizole (MET), metronidazole (METRO), nicotine (NIC), propranolol (PRO), sulfamethoxazole (SUL), trimethoprim (TRI), valsartan (VAL), and venlafaxine (VEN) (Table 1). The presence of 10 TPs was also evaluated: 4-acetamide antipyrine (AAA), atenololic acid (ATEAC), carbamazepine epoxide (CBZPOX), cotinine (COT), 4-dimethilaminoantipyrine (DAA), 4-formylaminoantipyrine (FAA), 4-hydroxy-diclofenac (HDIC), 3-metoxy-acetaminophen (MACE), N4-acetylsulfamethoxazole (N4ACE), and o-desmethylvenlafaxine (OVEN) (Table 1). The 7 TMs selected were arsenic (As), cadmium (Cd), chromium (Cr), copper (Cu), nickel (Ni), lead (Pb), and zinc (Zn). All reagents used were of analytical grade and were obtained from Merck (Darmstadt, Germany), Sigma-Aldrich (Burlington, MA, USA), Scharlau (Sentmenat, Barcelona, Spain), and Fisher Scientific (Hampton, NH, USA). More details about chemicals (isotope-labelled standards) and reagents used in sample analyses, and about stock preparations, can be found in the Supplementary Materials.

### 2.2. Agricultural Soil

A representative parcel cultivated with maize was selected in an agricultural area, in the Community of Madrid (Spain), regularly irrigated with water from the Jarama River, of which one of the major tributaries is the Manzanares River [41]. In the field, approximately 65 kg of bulk soil was sampled from the uppermost horizon (0–30 cm) at different points along the main diagonal of the parcel using a manual auger. The composite soil sample was air-dried, gently crushed, passed through a 2 mm sieve, and stored in high-density polyethylene (HDPE) buckets prior to physico-chemical analysis and bioassay experiments. The collected soil has a moderately alkaline pH (8.5) due to its calcareous nature (6.3% equivalent CaCO_3_-ECC-), with a silty loam texture (28% sand, 62% silt, 10% clay), and 2.1% of organic matter (OM) content, as is usual for agricultural soils in the Mediterranean area.

### 2.3. Experimental Setup

The bioassay experiment was carried out in pots with lettuce (*Lactuca sativa* L.) cv Summer Wonder, a variety well adapted to the Mediterranean climate. Lettuce seedlings were purchased from a local organic supplier (La Troje, Madrid, Spain). Each pot (15 cm diameter; 176.7 cm^2^ area) was filled with 1.45 kg of 2 mm soil mixed with 0.91 kg of 6–9 mm gravel (acid washed) (1:1.6 ratio, gravel/soil). A layer of 0.55 kg of 20–40 mm pebbles at the base was used to promote water drainage. Before transplanting, seedlings’ roots were gently washed with tap water and rinsed with Milli-Q water to remove the substrate. Successively, one lettuce seedling was planted per pot, and a total of twelve pots were randomly grown during 45 days in a growth chamber under controlled conditions (20 °C temperature, 40% humidity, 18/6 h light/dark cycles, and 248 ± 3 µmol m^−2^ s^−1^ photosynthetic active radiation). To prevent cross-contamination from drainage water, each pot was placed on a saucer. A pesticide application was made at the beginning of the bioassay to control aphids.

The synthetic water used for irrigation resembled the chemical composition of the real irrigation water at the field site in terms of major ions and organic carbon content. A supplement of NPK nutrient (2:1:3 ratio) was also added to promote plant growth. The following reagents (in mg L^−1^) were used to obtain the desired composition of synthetic water (SW): 80 CaCl_2_, 5 MgCl_2_, 50 NaCl, 25 NH_4_Cl, 150 CaSO_4_, 100 MgSO_4_, 19 K_2_HPO_4_, 2 KHCO_3_, 200 NaHCO_3_, 2 (NH_4_)_2_CO_3_, 40 KNO_3_, 25 NH_4_NO_3_, 2 peptone, and 11 meat extract. During the 3 days prior to the beginning of the experiment, all pots were irrigated with this SW. Afterwards, the SW was spiked with a mixture of (i) 7 TMs (100 µg L^−1^), (ii) 22 Phs (10 µg L^−1^), (iii) 7 TMs (100 µg L^−1^) + 22 Phs (10 µg L^−1^). A set of the experiment was used as a control (C, without spike). Each condition (C, TM, Ph, and Ph-TM) was done in triplicate (12 pots in total, 3 pots per condition). Taking into account the growth stage of the lettuce, the pots were irrigated twice per week with 250 mL (first 4-week growth stage) and 300 mL (last 2-week growth stage). In total, each lettuce received a total of 3.5 L throughout the growing period. All the material used was rinsed with ultrapure water, ethanol, methanol, and, finally, with ultrapure water.

### 2.4. Water, Plant, and Soil Sampling

Rhizon MOM soil moisture samplers (Rhizosphere Research Products, Wageningen, the Netherlands) were used to collect the interstitial water. The devices were horizontally placed in each pot at 5 cm depth in contact with the rhizosphere environment, and interstitial water samples were retrieved 1 time/week during 45 days. Each week, 24 h after the irrigation started, the vacuum was applied to the Rhizon MOM with a 5 mL syringe, and water was collected. Samples for the quantification of soluble TM contents were filtered through a 0.45 μm filter, acidified to a pH of 2–3 using HNO_3_, and stored in polypropylene (PP) tubes at −20 °C until analysis. Samples for Ph and TP determinations were collected in amber glass tubes and stored at −20 °C until analysis.

At the end of the bioassay (i.e., after 45 days) and prior to the harvesting, the height and aerial diameter of each plant were measured. Successively, the plants were harvested, leaves and roots were separated, and they were washed with tap water, followed by ultrapure water. Both tissues were gently dried with paper, and weighed. The tolerance index was calculated as a percentage of the leaf and root weights of plants grown in spiked conditions compared to plants grown in unspiked ones. Leaf lengths and numbers of leaves were also recorded. The leaves were further separated into external and internal ones. Finally, all tissues (external and internal leaves and roots) were frozen at −20 °C, lyophilized, powdered when needed, and stored in HDPE bottles until chemical analysis. The soil of each pot was wet-sieved at 2 mm to remove the gravel and quartered. An aliquot was stored at 5 °C until enzyme analyses, which were performed within 24 h after sampling. The rest of the soil samples were frozen, lyophilized, and stored in HDPE bottles until chemical analysis.

Water (irrigation and interstitial), plant tissues (external and internal leaves and roots), and soil samples were analysed for 7 TMs, 21 Phs (all spiked Phs except MET), 10 TPs, and 7 major element (ME) (Ca, K, Fe, Mg, Mn, Na, and P) contents. The anti-inflammatory MET has been described as very unstable under environmental conditions since it rapidly transforms into intermediates and TPs [41,45]. Therefore, this Ph was spiked in the irrigation water with the objective of exclusively addressing the fate of its TPs. The physico-chemical parameters of the synthetic irrigation water were regularly monitored in terms of pH, electrical conductivity (EC), redox potential, total organic carbon (TOC), chemical oxygen demand (COD), and dissolved major anions and cations.

### 2.5. Analytical Methods

#### 2.5.1. Water and Soil Physico-Chemical Analysis

Irrigation water samples were analysed for pH using a Crison pH 25 (UNE-EN-ISO 10523:2012), EC and redox potential using a Crison MM-41 (UNE-EN-27888:1994), TOC by combustion and infrared spectrophotometry (UNE-EN-ISO 5814, Shimadzu TOC-VCSH analyser with an autosampler, ASI-V), COD by the dichromate method (UNE-77004:2002) using a Merck (Darmstadt, Germany) Spectroquant TR420 and Spectroquant Pharo 100 Spectrophotometer, and dissolved major anions and cations using a 930 Compact Ion Chromatography Flex (autosampler 858 Professional Sample Processor) coupled to a Titrando 809 (autosampler 814 USB Sample Processor, Darmstadt, Germany) for HCO_3_^−^ ions (Metrohom, Herisau, Switzerland).

Prior to the bioassay experiment, the soil was analysed for pH (UNE-ISO 10390:2012), OM by the loss-on-ignition (LOI) method at 360 °C for 24 h, particle-size distribution by the Bouyoucos method (UNE 103102:1995), and ECC by the acid neutralisation method.

#### 2.5.2. Pharmaceutical and Transformation Product Analysis by LC-MS/MS

The quantification of Phs and TPs in water, soil, and lettuce samples was carried out using a liquid chromatograph (LC) (1200 series, Agilent Technologies, Santa Clara, CA, USA) coupled to a triple quadrupole (MS/MS) mass spectrometer (6495A, Agilent Technologies), equipped with an electrospray ionisation (ESI) interface. Ions were generated using an electrospray ion source with Agilent Jet Stream Technology, in positive and negative mode. Chromatographic separations and instrumental parameters are summarised in Table S1. The sensitivity of the instrumental method was estimated by establishing the limit of detection (LODi) (S/N 3) and the limit of quantification (LOQi) (S/N 10). The linearity for each compound was established from the corresponding LOQi level to a maximum concentration of 20 μg L^−1^, using external standards over two concentration ranges: for low levels (100 ng L^−1^) and high levels of quantification (20 μg L^−1^). The standard regression line was obtained as the mean of three injections of each calibration point, which had a regression coefficient (R^2^) *>* 0.99. Information on the retention times (t_R_), the multiple-reaction monitoring (MRM) transitions, and the collision energy (EC) for the different compounds is provided in Table S2.

Irrigation and interstitial water samples were analysed by direct injection after centrifugation (5 min at 13,000 rpm) and dilution according to instrumental requirements. Soil and lettuce (leaves and roots) samples were subjected to extraction treatment following the conditions detailed in Meffe et al. [41] with some minor modifications. Freeze-dried soil samples (1 g) were weighed and mixed with 200 μL of a solution of isotope-labelled internal standards of 40 μg L^−1^. Two sequential extractions (under basic and acidic conditions) based on EPA Method 1694 were performed in parallel for the analysis of all target compounds. The aqueous solutions resulting from both extractions were subjected to a solid-phase extraction (SPE) process using Oasis HLB cartridges (1 g, 20 mL). Finally, the organic extracts were evaporated, reconstituted in 4 mL of MeOH:H_2_O (10:90, *v*/*v*) and centrifuged prior to LC-MS/MS analysis. Freeze-dried leaf (0.5 g) and root samples (0.25 g) were weighed, and a solution of isotope-labelled internal standards of 60 μg L^−1^ was added (100 μL and 50 μL for leaf and root samples, respectively). A double extraction was performed by adding 15 mL of MeOH, and sonication in an ultrasonic bath for 15 min was carried out. The extracts were centrifuged, and the supernatants were mixed. For leaf samples, supernatants were evaporated to a final volume of 1 mL and diluted to 200 mL with ultrapure water containing 30 mg L^−1^ of EDTA. Aqueous solutions were passed through OASIS HLB SPE cartridges (1 g, 20 mL). The organic extracts were evaporated to dryness and reconstituted in 1 mL of MeOH/water (10:90, *v*/*v*). Finally, leaf extracts were centrifuged and diluted 1/10 prior to LC-MS/MS analysis. In the case of root extracts, two aliquots (5 mL) of supernatant were taken, diluted to 50 mL with ultrapure water (one of them at 8–9 pH value), and subjected to an SPE process using OASIS HLB cartridges (200 g, 6 mL). The organic extracts were evaporated to dryness and reconstituted in 0.5 mL of MeOH:H_2_O (10:90, *v*/*v*). Finally, root extracts were centrifuged and analysed by LC-MS/MS. Detailed sample treatment protocols are presented in the Supplementary Materials.

The extraction efficiency for target compounds was evaluated in soil and plant (leaves and roots) matrices. The accuracy and precision (*n* = 3) of the method were evaluated as recovery (R) and relative standard deviation (RSD) percentages using fortified samples. Recoveries (%) and RSDs (%) in solid samples were evaluated at concentration levels of 8 ng g^−1^ (for soil) and 12 ng g^−1^ (for root and leaf samples). Analytical quality parameters (LODi, LOQi, methodological recoveries, and RSD) are provided in Table S3. In order to control possible sample losses and matrix effects, isotope-labelled internal standards were used and validated recovery percentages were applied to determine methodological quantification limits (MQLs) and to achieve a reliable quantification.

#### 2.5.3. Trace Metal, Metalloid, and Major Element Analysis by ICP-MS

For ICP-MS analysis, the pH of water samples was adjusted to <2 using HNO_3_ (69%), and then samples were filtered through 0.45 µm PVDF syringe filters; Vidrafoc (Barcelona, Spain). The quantification of TM levels was carried out by using a quadrupole ICP-MS 7700x series from Agilent Technologies (Santa Clara, United States). The mass calibration of the ICP-MS instrument was tuned daily with a solution containing 1µg L^−1^ of Ce, Co, Li, Mg, Tl, and Y in 1% (*v*/*v*) HNO_3_. Plasma conditions and acquisition parameters for ICP-MS are given in Table S4. TM speciation modelling in interstitial water was performed using the Visual Minteq model ([46], v. 4.0).

Prior to the analysis of TMs, soil and plant samples were digested. The latter matrix was additionally analysed for major elements (MEs). Aliquots of 250 mg of each sample type were subjected to a treatment by microwave digestion at 190 °C for 10 min (Microwave Digestion System Ethos One, Milestone, Bergamo, Italy) with a mixture of HNO_3_:H_2_O_2_ (4:1). The digested extracts were filtered using 0.45µm PVDF syringe filters and diluted according to experimental requirements before analysis by ICP-MS following the operation conditions used for water samples. Analytical quality parameters (LODi, LOQi, and MQL) are summarised in Table S5.

#### 2.5.4. Soil Enzyme Activities

Soil extracellular hydrolytic enzyme activity was evaluated in soils after 45 days of irrigation. Four representative enzyme activities of the main biogeochemical cycles (C, N, P, and S) were selected and quantified according to ISO/TC 190/SC 4 N 422 2016: β-galactosidase (BGAL), urease (URE), phosphatase (PHOS), and aryl sulfatase (ARYS), respectively. Briefly, for each soil sample, soil suspensions were prepared in triplicate with Milli-Q water (1:6.25 *w*:*v*) and stirred at 250 rpm for 10 min. Then, each suspension was incubated in triplicate with substrates in 96-well plates, as follows: 4-nitrophenyl galactopyranoside (0.02 M, 3 h at 37 °C for BGAL), urea (0.4 M, 3 h at 25 °C for URE), 4-nitrophenyl phosphate (0.05 M, 30 min at 37 °C for PHOS), or 4-nitrophenyl sulphate (0.025 M, 4 h at 37 °C for ARYS). For each sample, triplicate suspensions without substrate were considered as a control. The reaction was stopped with 0.5 M CaCl_2_ and 0.1 M Tris pH 12 (for BGAL, PHOS, ARYS), or HACH (ammonium salicylate and ammonium cyanurate) (for URE), centrifuged (1.300× *g*, 5 min), and then supernatants were transferred into new 96-well plates. The 4-nitrophenol (BGAL, PHOS, ARYS) and NH_4_^+^ (URE) released were measured at 405 and 650 nm, respectively, with a UV–visible 96-well microplate spectrophotometer, Multiskan (Thermo Fisher Scientific). All reagents were acquired from Sigma-Aldrich. Activities were expressed in mU g^−1^ dry soil. One U of activity was defined as the amount of enzyme that catalysed 1 µmol of substrate in 1 min.

### 2.6. Contaminant Uptake and Translocation and Health Risk Assessment

To evaluate uptake and translocation processes, the soil-to-root bioconcentration factor (BCF_soil_) and the root-to-leaves translocation factor (TF) were calculated as follows: (i) BCFs as the ratios between the concentrations of Phs, TPs, or TMs, in lettuce roots (in dry weight, DW), and their corresponding soil content, and (ii) TFs as the ratios between the concentrations of Phs, TPs, TMs, and MEs, in DW, in lettuce leaves and in roots.

To evaluate the potential health risk derived from the consumption of lettuce containing Phs, TPs, and TMs, both the Hazardous Quotient (HQ)-based method (for Phs, TPs, and TMs) and the threshold of toxicological concern (TTC) approach (for Phs) were applied. The HQ was calculated as the ratio between the Estimated Daily Intake (EDI, μg kg^−1^ body weight (BW) day^−1^) and the acceptable daily intake (ADI), for each Ph and TP, or the reference dose (RfD), for each TM, and this was in turn divided by a BW of 70 kg (European adults, according to the European Food Safety Authority (EFSA) Scientific Committee [47]). In the case of TMs, this approach was also applied for toddlers (BW: 12 kg, [47]) and children (BW: 24 kg, [48]). Oral RfD values of TMs are the maximum tolerable daily intake by oral administration (μg kg^−1^ BW day^−1^). The oral RfD values used were 300, 400, 1500, 0.3, 20, and 3.5 μg kg^−1^ BW day^−1^ for Zn, Cu, Cr, As, Ni, and Pb, respectively [49]. In the case of Cd, 0.36 μg kg^−1^ BW day^−1^ was used, which comes from dividing by 7 the value of 2.5 μg kg^−1^ BW week^−1^ proposed by the EFSA [50]. The EDI was calculated as the daily intake (DI, g day^−1^) of lettuce, which is 17 g day^−1^ for adults, 6 g day^−1^ for children, and 1 g day^−1^ for toddlers, as reported by The Global Individual Food Consumption Data Tool of the FAO/WHO [51], multiplied by the Ph, TP, or TM concentration (average of external and internal leaves, mg kg^−1^ wet weight, WW). In the case of As in internal leaves, LOQ/2 was used. The ADI of each Ph and TP was calculated as the ratio between the daily minimum therapeutic dose (MTD) for adults (mg d^−1^) by a safety factor of 1000. The safety factor takes into account differences in human responses, the potential sensitivity of population subgroups, and the lowest daily therapeutic dose not being at a level that represents no effect [52]. The MTDs for Phs were retrieved from different databases and publications [52,53,54]. Codeine’s daily MTD was considered according to the European Medicines Agency (EMA) [55]. The ADI value for NIC was retrieved via data from the EFSA [56]. In the case of TPs, the ADI was set as equal to the daily MTD of the parent compound. A Hazard Index (HI) was also calculated as the sum of the HQs for all Phs, TPs, or TMs detected in lettuce leaves.

The TTC approach is usually used when there are limited toxicity data [57,58,59] as a tier 0 assessment to highlight the necessity for specific toxicity analysis when the consumption of substances, in this case, Phs, is higher than the TTC value. The approach is based on the comparison of a substance’s molecular structure with structural alerts [60] to assign each Ph to the corresponding category (Class I, II, III) through the Cramer classes decision tree [61] implemented in the software Toxtree ([62], v. 3.1.0). In our study, we used the revised Cramer decision tree. The TTC values of 30.0, 9.0, and 1.5 μg kg^−1^ day^−1^ were defined for Class I, II, and III, respectively [63]. According to Kroes et al. [60], a TTC value of 0.0025 μg kg^−1^ day^−1^ was used for potential genotoxic substances (i.e., carbamazepine epoxide). The daily consumption (DC, kg day^−1^) by an adult, a child, or a toddler to reach the TTC was calculated with Ph and TP concentrations, by averaging values detected in both external and internal leaves (C_avg_, ng g^−1^ WW) and applying the formula (TTC*BW)/C_avg_. Finally, the DC was compared with the DI of lettuce.

### 2.7. Statistical Analysis

The significance of the differences among conditions (C, TM, Ph, and Ph-TM) in mean values of each—Ph, TP, and TM concentration in interstitial water and soil; Ph, TP, TM, and ME amounts in plant tissues (roots, external leaves, and internal leaves); plant tissue biomass and other ecophysiological parameters; BCF and TF; as well as BGAL, URE, ARYS, and PHOS enzyme activity—was investigated by means of one-way analysis of variance (ANOVA) using a post hoc test (Tukey). The homogeneity of variances was verified by the Levene test. When only two conditions were compared (e.g., Ph and Ph-TM), a Student’s *t*-test was employed to investigate the significance of differences in the mean values of the contaminant concentration and/or content. Correlation between Ph concentrations in interstitial water, soil, and plants, for each condition, was assessed with Pearson analysis. These analyses were done using the open source software PSPP v.2.0.1 (Free Software Foundation, Inc., Boston, MA, USA)

## 3. Results and Discussion

### 3.1. Pharmaceuticals and Transformation Products

#### 3.1.1. Phs and TPs in the Soil–Water Interface

Pharmaceuticals are detected in the interstitial water throughout the 45 days of irrigation in a wide range of concentrations. As expected, most of the target Phs and TPs are not detected in the interstitial water under unspiked conditions (C and TM) (Table S6). However, 11 out of 22 target Phs are found to be either below the LOQ or to rarely appear with very low concentrations. These include VEN, FLE, ATE, TRI, COD, PRO, CIT, ENA, NIC, and CLA, as well as the TP AAA. Furthermore, the TPs MACE, DAA, HDIC, and OVEN are never detected in the interstitial water under any condition (spiked or unspiked). Some drugs (SUL, CBZ, and CBZPOX) are found as a consequence of their background soil contents, attributed to their high persistence (see Section 3.1.2). Specifically, concentrations in the interstitial water under unspiked conditions range as follows: SUL from <LOQ to 170.2 ng L^−1^, CBZ from 32.9 to 223.2 ng L^−1^, and CBZPOX from 21.6 to 36.4 ng L^−1^. The persistent behaviour of SUL, CBZ, and CBZPOX agrees with the results obtained in a previous field study investigating natural attenuation processes during infiltration through the same soil used in these bioassays [41]. Other Phs and TPs are occasionally detected in the interstitial water in unspiked conditions (C and TM) as a result of desorption from the soil. Indeed, ACE (69.1 ± 16.1 ng L^−1^) and FAA (28.8 ± 2.7 ng L^−1^) are detected only in the first 2 weeks of the experiment.

**Figure 1:**
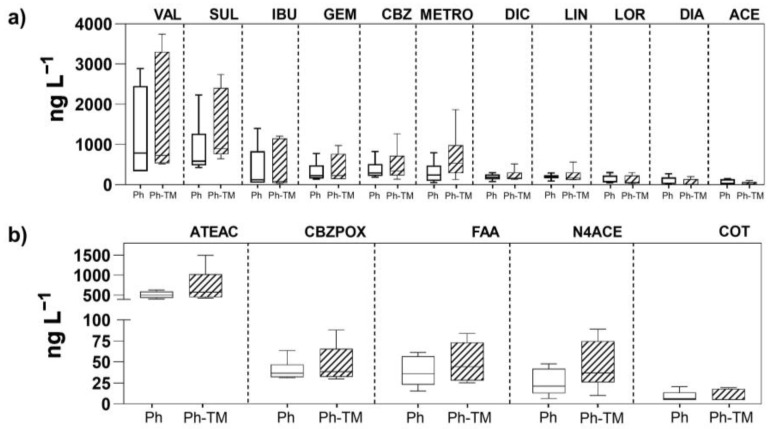
Pharmaceutical (**a**) and transformation product (**b**) concentrations in the interstitial water from Ph and Ph−TM conditions over the 45-day irrigation period. The boxplots show the lower quartile, the median, and the upper quartile, with whiskers extending to the most extreme data point. Compounds are represented in order of decreasing concentrations.

Figure 1 shows Ph and TP concentrations in the interstitial water under Ph-spiked conditions (Ph and Ph-TM). Results indicate that 21 Phs and 5 TPs are detected in the interstitial water, regardless of whether they were in combination or not with TMs. Although dissimilarities in concentration ranges are observed between the two conditions, these are not statistically significant. The data on the concentration of Phs and TPs in the interstitial water correspond to the set of irrigation events throughout the 45 days of the bioassay. The wide range of variation in each condition is due to the fact that the concentration of Phs and TPs is highly time-dependent, as discussed below. This wide range of variation makes comparisons difficult. On the contrary, significant differences are observed in the concentrations of TMs in the presence and absence of Phs, and further discussion will be provided in Section 3.2.1. The interaction between Phs and TMs can greatly influence their behaviour in the soil–water interface. However, when either the Ph or TM has a high intrinsic affinity for binding to soils, the effect exerted by the co-occurrence of TMs and Phs may be limited for the compound with the higher sorption affinity. Focusing on concentration levels, Figure 1a shows the concentrations of the Phs in the interstitial water with values ranging from 25 ng L^−1^ (ACE) to 3744 ng L^−1^ (VAL). Most of the quantified Phs belonging to this group (Figure 1a) are predominantly in a neutral or anionic state and have a hydrophilic nature (log D_ow_ < 3) (except benzodiazepines), and these properties well justify their occurrence in the interstitial water. VAL and SUL exhibit the highest concentrations compared to all other Phs, which represent about 12% of the spiked concentrations in the irrigation water. VAL is a very mobile compound (log D_ow_ 0.2) with a fast dissipation in soils [64,65]. In previous studies involving this soil, it was determined that VAL does not exhibit sorption onto the soil matrix [41]. Therefore, it is expected to be present in higher contents in the interstitial water. The sulphonamide antibiotic SUL is a hydrophilic compound (Log D_ow_ −0.15) and negatively ionised at the interstitial water pH (8.7). According to the literature, desorption of this antibiotic is enhanced by the increase of pH (from 4 to 9) [66]. Such conditions are altogether responsible for a pronounced mobility of SUL in soils and justify its higher concentration levels in the interstitial water. As concluded by Meffe et al. [41], it is plausible to think that, after bypassing the most reactive soil layer, drugs such as VAL and/or SUL may reach groundwater.

Five out of the eleven target TPs are found in the interstitial water with levels above the LOQ (Figure 1b). ATEAC was detected with the highest concentration (399–623 ng L^−1^ Ph and 426–1495 ng L^−1^ Ph-TM). The rest of the TPs (CBZPOX, FAA, N4ACE, and COT) occur with levels below 100 ng L^−1^ in both conditions. Two different behaviour patterns are observed for TPs. Thus, some TPs are present in lower concentrations than their parent compounds (N4ACE and CBZ), while others exhibit higher concentrations (ATEAC, COT, and FAA). It is important to mention that certain TPs are detected in the irrigation water (N4ACE and ATEAC both detected but at <LOQ, and COT up to 296 ng L^−1^), indicating their formation before being in contact with the soil. As a rough approximation of the transformation process occurring during the bioassay, a transformation ratio (TR) has been calculated, taking into account the concentration of the parent compound in the irrigation water and the concentration of the corresponding TP in the interstitial water. Thus, the TRs of SUL into N4ACE are 0.3% and 0.4%, and those of CBZ into CBZPOX are 0.1% and 0.2% in Ph and Ph-TM conditions, respectively. As discussed in Section 3.1.2 and Section 3.1.3, N4ACE is not retained in the soil, and it is not taken up by the plant; therefore, the calculated values of the TR should likely be attributed to degradation processes occurring in the interstitial water. Instead, CBZPOX is ubiquitous among the investigated matrices (interstitial water, soil, and plant tissues), and further degradation, plant uptake, and sorption onto soil colloids can not be ruled out. By contrast, the average concentration of ATEAC exceeds that of its parent compound ATE in the interstitial water. Its TR is 4.9% in Ph and 7.3% in Ph-TM conditions. In agricultural soils, ATE rapidly undergoes hydroxylation to form ATEAC, which is more persistent than its parent compound [67]. The greater presence of ATEC under the co-occurrence of Phs and TMs would indicate that the presence of TMs favours either the transformation of ATE into ATEAC, or the persistence of the formed ATEAC. COT, similarly to CBZPOX, is also ubiquitous in target matrices. However, its parent compound presents values that are always below the LOQ; therefore, the calculation of its TR was not possible. Among the three target TPs of MET, FAA and AAA are detected, although only FAA appears with concentrations above the LOQ (from 15 to 70 ng L^−1^). Previous studies reported that these two TPs are more stable and persistent than the intermediates AAA and DAA [3,41,45,68].

**Figure 2:**
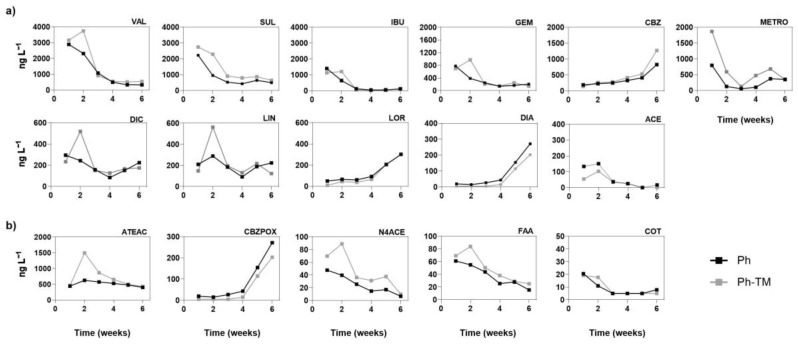
Temporal trends of pharmaceutical (**a**) and transformation product (**b**) concentrations in interstitial water from Ph and Ph-TM conditions over the 45-day irrigation period. Compounds appear in order of decreasing concentrations.

Figure 2 plots the temporal trend of the concentrations of Phs and TPs in the interstitial water throughout the 45 days of the bioassay under Ph-spiked conditions (Ph and Ph-TM). The data indicate three different behaviours: (i) Phs with a decreasing concentration trend; (ii) Phs with an increasing concentration trend, and (iii) Phs with a mixed temporal trend (i.e., a decrease followed by an increase after the 4th week). The Phs VAL, SUL, IBU, GEM, and ACE exhibit a reduction of between 74 and 91% when compared to the initial concentration in the interstitial water (at week 1), and such a reduction reaches a practically total dissipation of the Phs at week 6 (close to 100%). Conversely, the concentrations of CBZ, LOR, and DIA in the interstitial water increases up to 14 (Ph) or 41 times (Ph-TM) the levels initially detected at week 1. As a result, the pharmaceutical composition in the interstitial water is substantially different at the beginning and at the end of the irrigation period, regardless of the presence of TMs. It is interesting to note that from week 1 to week 6, CBZ and benzodiazepine concentrations increase from 165 ng L^−1^ to 1137 ng L^−1^ (CBZ), from <LOQ to 356 ng L^−1^ (LOR), and from <LOQ to 296 ng L^−1^ (DIA) on average under Ph and Ph-TM conditions. Although results show that the concentration of most drugs decreases over time in the interstitial water, attention should be paid to those Phs (CBZ and benzodiazepines) prone to desorb from soil to water, since they can be taken up by crops or they can infiltrate towards deeper levels, reaching groundwater resources [69,70,71]. Concerning TPs, the temporal trend of the concentrations of CBZPOX and N4ACE in the interstitial water follows the same pattern as their parent compounds CBZ and SUL. The TP N4ACE has attracted significant attention in the literature due to its frequent occurrence in different environmental samples [72], sometimes exceeding the concentration of its parent compound [73]. The time-variable trends of CBZ and CBZPOX are in agreement with the results of Fenet et al. [70], who suggest that the affinity of both Phs for soil decreases with increasing concentrations. Finally, ATEAC, FAA, and COT concentrations decreases over time. However, comparisons with their respective parent compounds could not be carried out since these are not detected in the interstitial water, or they are present in concentrations below the LOQ. 

#### 3.1.2. Ph and TP Accumulation in the Soil

Several Phs and TPs are detected in the unspiked soil (C) as a consequence of the continuous irrigation with surface water, which is affected by the discharge of WWTP effluents. Thus, 11 Phs and 4 TPs are found in the unspiked soil with contents according to the following order: FLE (12.3 ng g^−1^) > VEN (9.3 ng g^−1^) > CBZ (5.8 ng g^−1^) > ATEAC (2.3 ng g^−1^) > CLA, PRO, CIT, SUL, LOR, DIA, CBZPOX, COT, and TRI (<1.2 ng g^−1^). Some Phs and TPs are detected but not quantified: ACE, DIC, GEM, IBU, LIN, METRO, N4ACE, NIC, OVEN, and VAL. The rest of the compounds were never detected, and the majority of them are TPs. Similar results are observed in the soil where only TMs were spiked. The contents of the Phs and TPs quantified in unspiked soils (C and TMs conditions) are shown in Table S7.

**Figure 3:**
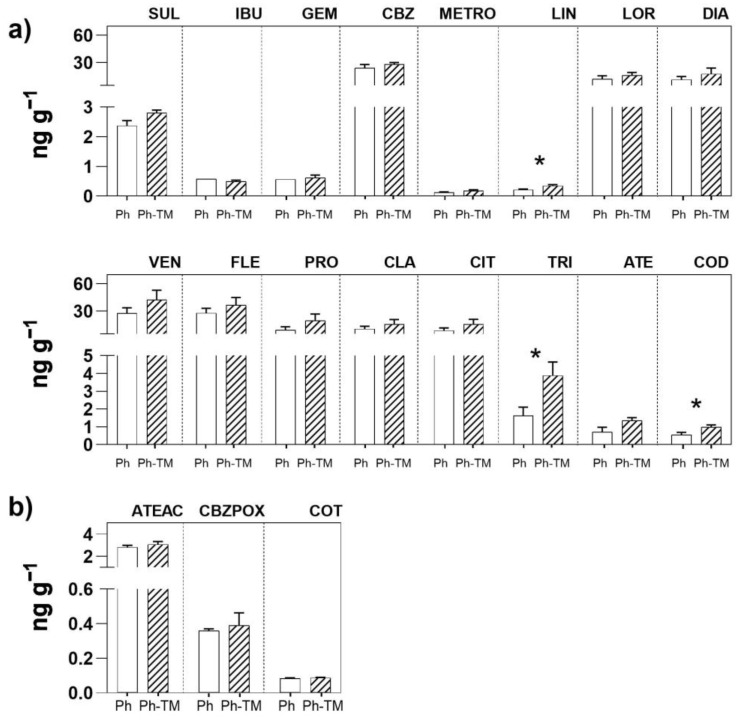
Total average soil contents of pharmaceuticals (**a**) and transformation products (**b**) (dry weight) from Ph and Ph-TM conditions after the 45-day irrigation period. Error bars are standard deviation. For comparative purposes, compounds appear in order of decreasing concentrations in interstitial water. Differences between Ph and Ph-TM conditions were calculated by a Student’s *t*-test. * indicates significant differences at *p* < 0.05. In the case where there are no significant differences, nothing is indicated.

As expected, Ph soil contents under spiked conditions (Ph and Ph-TM) are significantly higher than Ph soil contents under unspiked conditions. The only exception is represented by COT, where no significant differences are observed between spiked and unspiked conditions. The contents of the 17 Phs and four TPs quantified in Ph-spiked soils (Ph and Ph-TM) are shown in Figure 3. Pharmaceuticals and TPs occurring with the highest contents in Ph-TM-spiked soils are VEN, CBZ, and FLE (28–43 ng g^−1^ range), followed by PRO, DIA, LOR, CLA, CIT (16–20 ng g^−1^ range), TRI, ATEAC, SUL, ATE (1–4 ng g^−1^ range), and the rest < 1 ng g^−1^. Similar ranges are observed in the soil where only Phs were added. FLE and VEN, being positively charged Phs, tend to be attracted to negatively charged soil colloids, such as the OM and clay minerals. This attraction facilitates their sorption via cation exchange processes, resulting in a high sorption capacity and stability in soils [41,74,75]. The polar and uncharged antiepileptic drug CBZ presents a weak sorption onto soil (distribution coefficient, K_d_, ranging from 4.2 to 20 L kg^−1^) but a high persistence (half-life, T_1/2_, ranging from 125 to 233 days) [65,76]. The soil contents of most target Phs is not affected by the presence of TMs, with the exception of LIN, TRI, and COD, whose contents is significantly higher under Ph-TM conditions. A similar behaviour is observed for the rest of the Phs and especially for SUL, VEN, FLE, PRO, and ATE. However, in these cases, differences in soil contents between Ph and Ph-TM conditions are not significant. Papaioannou et al. [16] found that certain Phs have a high capacity to interact with TMs, and these interactions can be synergistic or antagonistic. In our study, synergistic interactions increasing the content of Phs accumulated in the soil, which may also affect its mobility, have been found. Although the mechanisms of Ph-TM interaction have not been completely elucidated, cationic bridging and/or surface complexation fostered by the presence of TMs may favour Ph sorption onto soil [19] (see Section 3.2.1). Among Phs, antibiotic–metal complexes have been more widely studied. In particular, it has been reported that the antibiotic LIN effectively coordinates Cu ions [35,36], and TRI increases its sorption onto the soil in the presence of metal ions such as Cu, Zn, and Fe [77]. The interactions of LIN and TRI with TMs in soils deserve further studies since they may favour the dissemination of antibiotic resistance in the environment, as suggested by Khurana et al. [5]. On the other hand, the formation of metal complexes with non-steroidal anti-inflammatory (NSAID) ligands, such as COD, has been previously described, but only in in vitro studies [27].

Regarding the presence of TPs in the soil, ATEAC, CBZPOX, and COT are quantified in all conditions (except COT in TM-spiked soil). Similar to what is observed in the interstitial water, the content of ATEAC is higher than the content of its parent compound ATE. Although the contents of these TPs in the soil does not vary significantly between Ph and Ph-TM conditions, the TR calculated between ATEAC vs. ATE is different in the presence of TMs. Thus, ATEAC content is higher in Ph-TM-spiked soils (four times) than in Ph-spiked ones (two times). As observed in interstitial water (Section 3.1.1), results show that the biodegradation rate of ATE is enhanced in soils in the presence of TMs. As discussed in Section 3.5, certain microbial activity in soils is stimulated under Ph-TM conditions, which could explain this result. In contrast, regardless of the presence of TMs, the proportion of CBZPOX is very low, and it represents about 1.5% of the CBZ soil content, data that is in agreement with the low biodegradability of CBZ in soils. Moreover, CBZPOX presents a soil sorption affinity that is lower than that of the parent compounds, and, therefore, it has a higher mobility in soils. The lower sorption affinity of CBZPOX compared to its parental compound has been also observed by Paz et al. [71] and Malchi et al. [78]. The NIC content is <LOQ in all the conditions. Therefore, the COT content can not be solely attributed to the spiked NIC concentration, but also to transformation processes in Ph-spiked irrigation water (before irrigation, prior to contact with the soil) and to its background presence in the soil prior to the bioassay.

#### 3.1.3. Ph and TP Uptake and Translocation by Lettuce

Several Phs are taken up and then translocated by lettuce plants in unspiked soils (C). In order to overcome differences in biomass production, the total amounts of Phs and TPs taken up by lettuce were calculated by multiplying Ph and TP concentrations in each plant tissue by the corresponding tissue biomass. Thus, seven Phs and two TPs are quantified in the total biomass, with contents according to the following order: CBZ (55.8 ng) > VEN (11.6 ng) > CBZPOX (8.6 ng) > FLE (6.2 ng) > IBU, CIT, and DIA (<2.5 ng). Of these, CBZ, CBZPOX, VEN, and DIA are quantified in leaves and roots, IBU only in roots, and FLE and CIT only in external leaves. Apart from this group, most of the compounds are not detected or they appear with contents below the LOQ (i.e., NIC, COT, LIN, LOR, and PRO). Similar results are observed in the pots where only TMs were added. The contents of the Phs and TPs quantified in unspiked soils are in Table S8.

**Figure 4:**
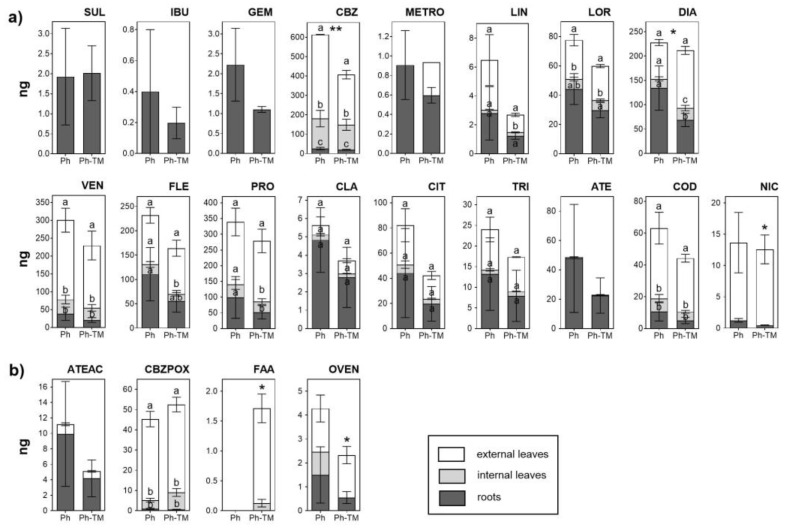
Total average amount of pharmaceuticals (**a**) and transformation products (**b**) in external leaves, internal leaves, and roots of lettuce plants grown in Ph and Ph-TM conditions after the 45-day irrigation period. Error bars are standard deviation. The absence of bars in external leaves of METRO and internal leaves of OVEN is due to only one replica being >LOQ. For comparative purposes, compounds appear in order of decreasing concentrations in the interstitial water. Different letters indicate significant differences among tissues for each condition at *p* < 0.05 after one-way analysis of variance. The amount of some compounds was lower than the LOQ in the internal leaves (NIC and OVEN) or in the roots (FAA); thus, significant differences for each condition were calculated by a Student’s *t*-test. Differences between Ph and Ph-TM conditions for each tissue were also calculated by a Student’s *t*-test. * and ** indicate significant differences at *p* < 0.05 and *p* < 0.01, for DIA and CBZ, respectively. In cases where there are no significant differences, nothing is indicated.

As expected, the amount of Phs taken up by plants under spiked conditions (Ph and Ph-TM) is significantly higher than in unspiked ones (C and TM). The contents of the 18 Phs and four TPs quantified in plant tissues in Ph-spiked pots (Ph and Ph-TM) are shown in Figure 4. Pharmaceuticals and TPs quantified with the highest contents in lettuce are CBZ, PRO, VEN, DIA, and FLE (160–400 ng range), followed by LOR, CBZPOX, COD, CIT, ATE, TRI, and NIC (10–60 ng range), and the rest < 5 ng. On the other hand, VAL, ENA, and the TPs N4ACE, AAA, DAA, HDIC, and MACE are never detected. DIC and ACE are below the LOQ. The soil-to-root BCF (BCF_soil_) values range from 0.3 to 121 in Ph conditions and from 0.2 to 42 in Ph-TM conditions. As discussed below, the formation of Ph-TM complexes is likely to prevent the uptake of both types of contaminants. Although a synergistic effect is observed in the decrease in BCF_soil_ by the co-occurrence of Ph and TM, this trend is not significant in the case of Phs. The BCF_soil_ generally exhibit positive correlations with the lipophilicity of neutral compounds. Lipophilic compounds are expected to partition onto root lipids, resulting in a greater uptake by roots [79]. To further investigate this relationship, the log BCF_soil_ in different plant tissues was plotted against the pH-adjusted octanol–water partition coefficient (log D_ow_) for Phs that predominantly exist in a neutral form at pH 8.7 (DIA, METRO, LOR, TRI, LIN, CBZ, and CBZPOX). Nonetheless, no correlation is found between log BCF_soil_ and log D_ow_ either. In contrast, cationic Phs (ATE, COD, and PRO) present the highest BCF_soil_ values, which indicates their higher tendency to be transported from soil to roots. It is worth mentioning that these Phs are found in the interstitial water either below the LOQ or with very low concentrations; therefore, their main source for plant uptake would be the soil. Indeed, when evaluating the group of Phs present in higher concentrations in the interstitial water (VAL, SUL, IBU, GEM, CBZ, and METRO), it is evident that their uptake by plants is generally limited, except for CBZ, which also present high levels in the soil. Pearson correlation analysis show a moderate positive correlation between Ph concentrations in the interstitial water and plants, whereas Ph concentrations in the soil and plants are highly positively correlated (Tables S9 and S10). Therefore, it can be concluded that even though Phs are sorbed onto the soil, desorption is not a limiting factor for their bioavailability, and therefore for their plant uptake [76]. It is important to note that those Phs whose soil content significantly increases in the presence of TMs (LIN, TRI, and COD) (see Section 3.1.2) do not present a greater uptake under Ph-TM conditions. Therefore, processes taking place in the soil under Ph-TM interactions could represent a limitation in the bioavailability of Phs to plants.

By differentiating between plant tissues, most of the compounds are quantified in leaves and roots, although the bioaccumulation is generally greater in the external leaves. Water flow caused by plant transpiration positively influences the translocation of compounds from roots to leaves [80,81]. The external leaves are older than the internal leaves; thus, they have been less protected from light for a longer time and, consequently, show higher transpiration, supporting the obtained results. In some cases, drugs are only quantified in the roots (SUL, IBU, and GEM), or in external leaves and roots (ATE, NIC, ATEAC, and OVEN). The translocation of Phs by crops is related to different factors, including plant physiology and compound properties. The physico-chemical characteristics of compounds such as ionic charge, lipophilicity, and molecular weight (MW) influence their ability to passively pass through the membrane of plant cells [82]. Data show that Ph uptake by lettuce occurs for neutral, cationic, anionic, and zwitterionic forms. However, anionic Phs remain in roots (SUL, IBU, and GEM), while neutral, zwitterionic, and cationic Phs are found in the leaves. This result is in agreement with the data reported in the literature. Thus, anions tend to accumulate in root tissues, while neutral and cationic species tend to translocate to aerial tissues via xylem driven by the transpiration stream [83].

The TF calculated to evaluate the Ph transport from roots to aerial tissues shows that the presence of TMs affects the Ph bioaccumulation pattern. In general, an increase in TF values in the presence of TMs is observed (except for CBZ and LIN), although it is only statistically significant for NIC and DIA. According to their TFs, Phs can be divided into three groups: (i) Phs with TFs > 1 in both conditions (Ph and Ph-TM), (ii) TFs < 1 in Ph and TFs > 1 in Ph-TM, and (iii) Phs with TFs < 1 in both conditions. The first group includes CBZ, CBZPOX, NIC, VEN, COD, PRO, OVEN, and CIT. Among these, the neutral compounds CBZ and CBZPOX have the highest tendency to be translocated; the other Phs with lower translocation capacities are present in their cationic form. This can be explained by the fact that cationic compounds can be trapped in the apoplast or root vacuoles (pH ~5), resulting in a reduced concentration available for translocation to aerial parts [84]. All these compounds are expected to exhibit a great uptake and translocation due to their physico-chemical properties: log *D*_ow_ values in the range of 0.9–2.8, MW < 350 Da, H-bond donors < 2, and H-bond acceptors < 4 [85]. Generally, Phs with moderate log *K*_ow_ values have a high potential to be translocated in plants. Thus, organic compounds with log *K*_ow_ values ranging from 0.5 to 3 may behave simultaneously as hydrophilic and lipophilic, which facilitates their transfer to the aerial organs of the plants by translocation through the lipid bilayer of cell membranes [86]. Results show that CBZPOX, with a log *K*_ow_ ~1.9, present the highest TF. This evidence agrees with the result obtained by Briggs et al. [87], who defined that the maximum translocation is observed at a log *K*_ow_ of ~1.8. The second group include DIA, FLE, and TRI, and exhibit an enhanced translocation in the presence of TMs. However, this trend is only significant in the case of DIA. FLE and TRI are mainly detected in the soil matrix, and not in the interstitial water (<LOQ). Both have an intermediate log D_ow_ (2.2 and 1.3, respectively) and mostly exist as cationic molecules at pH 4–6. Although some properties coincide with those of other translocation-prone Phs, these results may be interpreted considering the mutual influence of additional factors, such as the competition between Phs, which may have a role in the translocation. Moreover, FLE translocation may be limited by its MW (414.1 Da). The higher root accumulation of DIA (present as a neutral compound) should be related to hydrophobic sorption, and is influenced by its log *D*_ow_ [84,88]. Nevertheless, as mentioned above, the translocation of DIA show an increasing trend in the presence of TMs. The formation of complexes among TMs and DIA can not be evoked to explain the enhanced translocation since Ph-TM complexes are most likely to hamper this process. Also, as described below (Section 3.2.3), TM translocation is generally decreased in the Ph-TM condition. The presence of TMs may be enhancing the sorption mechanisms of certain Phs over others in the roots, which could increase the DIA availability for translocation. The third group includes LOR, ATE, ATEAC, and CLA. LOR is a neutral compound with the highest log D_ow_ value of all the studied Phs, which may limit its translocation from roots, since higher lipophilicity may allow faster diffusion between lipid bilayers but may impede translocation in the cell wall or the cytosol [82]. The limited translocation of ATE and ATEAC is probably related to their high hydrophilicity (log *D*_ow_ of ~−0.3 and −1.3, respectively). This result agrees with previous studies that have reported that compounds with very low log K_ow_ were not efficiently translocated from roots due to the lack of lipophilicity. ATE and ATEAC may cross cell membranes more slowly at the Casparian strip barrier, so their partitioning into the xylem sap is not optimal [89,90]. The limited translocation of CLA may be related to its high MW (747.5 Da) since the uptake and the movement may be restricted by the cell wall. While some authors consider that organic compounds with MW < 1000 Da are easily taken up by plants [91], it has been documented that the translocation for large-sized Phs (MW > 400 Da) is limited [92]. With the exception of LIN, VAL, FLE and CLA, all target compounds have a MW < 400 Da.

Regarding the presence of TPs, ATEAC, CBZPOX, FAA, and OVEN were quantified in plant tissues. Unlike what is observed in the soil, COT is not detected. ATEAC mostly accumulate in roots, as does its parental compound ATE. However, in contrast to what is observed for the interstitial water and soil, the amount of ATEAC in roots is lower than that of ATE. On the contrary, higher amounts of ATEAC than ATE are quantified in external leaves (1.7 times). Nevertheless, this result should be taken with caution because the contents are very low (0.78 and 0.54 ng ATE and 1.24 and 0.91 ng ATEAC, for Ph and Ph-TM, respectively, on average). The higher concentrations of ATE and ATEAC in roots than in leaves were also documented by Kodesova et al. [67]. However, in contrast to our results, Kodesova et al. [67] reported larger concentrations of ATEAC than ATE in roots as well as a greater ATE mobility to leaves. The proportion of CBZPOX compared to CBZ in different plant tissues is low (ranging from 3 to 24%), but greater than what is observed in soils. These results are in line with those previously described, suggesting that CBZ is metabolised by plants [93], mainly in leaves, catalysed by cytochrome P450 enzymes [67,75,78]. It is interesting to note that while the amount of CBZ significantly decrease in external leaves in the presence of TMs, the amount of CBZPOX slightly increase, not being significant. In any case, it is observed that the Ph-TM interaction affects the metabolism of CBZ in the plant. OVEN accumulates in all tissues, as does VEN, but the proportion of OVEN with respect to VEN is very low (1–3%). Beyond the plant, OVEN is detected only in the soil and with concentrations <LOQ. Therefore, the metabolic processes of VEN (giving rise to TPs such as OVEN) are likely occurring in the plant, probably for cell detoxification. Exceptionally, FAA is only quantified in Ph-TM-spiked pots, and significantly more in the external leaves. This MET TP was detected in the interstitial water but not in the soil. Its uptake from the interstitial water and translocation to leaves could be explained by its intrinsic properties. FAA is a neutral compound with a hydrophilic nature; hence, its occurrence and translocation to leaves is expected [94,95]. As stated above, this behaviour may be attributed to competitive processes among Phs and TMs.

### 3.2. Trace Metals and Metalloid

#### 3.2.1. TMs in the Soil–Water Interface

**Figure 5:**
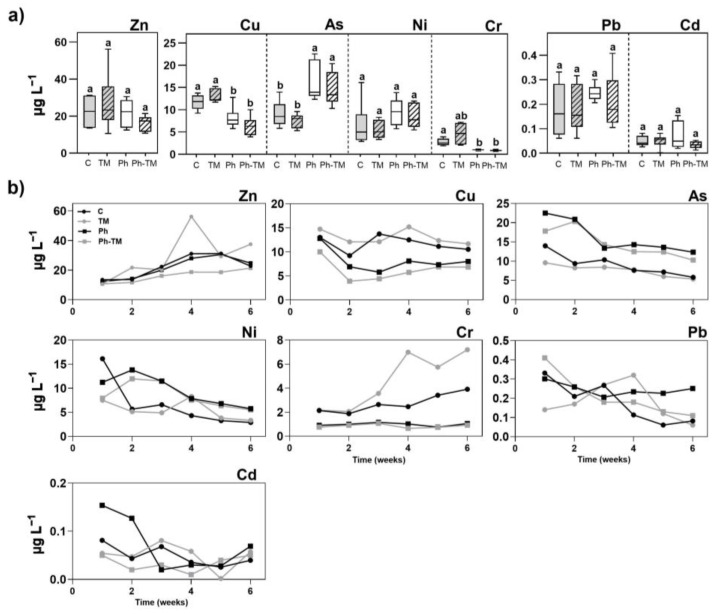
Trace metal and metalloid concentrations (**a**) and their temporal trends (**b**) in the interstitial water from C, TM, Ph, and Ph-TM conditions over the 45-day irrigation period. The boxplots show the lower quartile, the median, and the upper quartile, with whiskers extending to the most extreme data points. Trace metals and metalloid appear in order of decreasing concentration. Different letters indicate significant differences among conditions for each TM at *p* < 0.05 after one-way analysis of variance.

The seven selected TMs are detected in the interstitial water over the 45-day irrigation period, exhibiting a wide range of concentrations. Figure 5a shows the concentrations of TMs under C, TM, Ph, and Ph-TM conditions. As expected, TM concentrations in the interstitial water are quite low, and represent a low percentage with respect to the added concentration (100 µg L^−1^). In TM-spiked pots, the percentages, from the highest to the lowest, are as follows: 15% Zn, 12% Cu, 7% As, 6% Ni, 4% Cr, <0.4% Pb, and <0.06% Cd. Interestingly, TM concentrations in the interstitial water in TM-spiked pots are not significantly higher than in unspiked ones (C). Overall, Zn exhibit the highest concentrations, reaching values up to 62 µg L^−1^. Copper, As, Ni, and Cr are present in intermediate concentrations, below 25 µg L^−1^. Lead and Cd occur with the lowest concentrations, below 0.5 µg L^−1^. Given the calcareous nature of the study soil, TM precipitation is likely to occur, as carbonate complexes and hydroxy complexes, resulting in lower concentrations in the interstitial water of TMs such as Cu, Ni, Pb, and Cd [96]. Moreover, synergistic interactions between TMs may occur. At the soil pH of 8.5 and interstitial water pH of 8.7, the metalloid As can precipitate within oxyhydroxides, sulphates, or carbonates [97]. Thus, the solubility of As and Cu in the interstitial water of alkaline soils exhibit a notable reduction when these TMs coexist due to the precipitation of Cu-As phases [98]. However, precipitation is not the only mechanism responsible for the low TM concentration in the interstitial water. In addition to the carbonate fraction, sorption onto soil colloids (OM and clay) also plays an important role in calcareous soils, as described, for example, for Cu and Pb [39,99,100]. Also, desorption processes could occur, as described for oxyanions of As and Cr, which tend to be desorbed from Fe oxides with increasing pH owing to competition for sorption sites between the oxyanions and the OH^−^ ions [101]. Trace metal speciation was predicted using the Visual Minteq model. The measured pH (8.7) and TM concentration in the interstitial water were used as input data. Results can be summarised as follows: Ni and Cd mostly occur as free metal ions (92% and 96%, respectively), Cu, Cr, and Pb as hydroxyl species (97%, 100%, and 94%, respectively), Zn occur 79% as hydroxyl species and at 21% as free ions, and As speciate at 100% as oxoacids. No differences regarding TM speciation are observed in the presence or absence of Phs. The sorption of positively charged TM hydroxyl species is more prefer than that of free metal ions [101]; therefore, their low concentration in the interstitial water is explained.

In contrast to what is observed in the case of drugs, the concentrations of some TMs in the interstitial water are significantly different when Phs are spiked (Ph and Ph-TM conditions) with respect to unspiked assays (C and TM conditions). Two patterns are observed: (i) Cu and Cr concentrations are lower under Ph-spiked conditions than under unspiked conditions (30% and 52% Cu, 64% and 82% Cr, for Ph vs. C and Ph-TM vs. TM, respectively), and (ii) the As concentration is higher under Ph-spiked conditions than under unspiked conditions (79% Ph vs. C and 93% Ph-TM vs. TM). As mentioned above, pharmaceutical compounds can form complexes with Cu and Cr that could have greater affinity for soil colloids (clay and OM). Ma et al. [18] reported that heterocyclic N and sulphonamide N groups in the antibiotic SUL can form surface ternary complexes with Cu on ceramsite particles. These complexes contributed to the synergetic coadsorption of SUL and Cu onto adsorbents. Other drugs present in the interstitial water in the present study, such as LIN, TRI, COD, VAL, CBZ, DIA, and LOR, among many others, have a high affinity for Cu, tending to form Ph-Cu complexes, as well as for other cationic metals (e.g., Zn^2+^, Ni^2+^, Ca^2+^, Mg^2+^, etc.) [27,29,31,33,34,35,36,77]. In the same way, the complexes formed between Cr and drugs such as salicylic acid and its derivatives, metformin, and certain antibiotics (e.g., CLA), as well as their behaviour, have been widely described [25,26,28]. Research on the formation of Ph-TM complexes is extensive in the literature, covering clinical or pharmaceutical industry investigation, whereas studies about Ph-TM interaction in the environmental scenario are rather scarce. Another mechanism reported for alkaline soils is the cationic bridge between drugs (e.g., SUL, sulfadiazine, quinolones, and tetracyclines) and divalent metallic cations (Ca^2+^, Mg^2+^, Cu^2+^, etc.), increasing the drugs’ sorption onto soil colloids [19]. This mechanism is dose-dependent; there is competitive sorption between the drugs and TMs as the concentration of TMs increases, resulting in the decreased sorption of drugs. In the present study, Cu and Cr are present mostly as monovalent hydroxyl species (100% CrOH^+^, 50% CuOH^+^). Therefore, surface complexation seems to be the mechanism responsible for the decrease in Cu and Cr concentrations in water in the presence of Phs. It is most likely that both types of processes coexist, although one predominates over the other. The lack of significant differences found in the case of the drugs indicates that the interaction is probably due to a set of processes that do not result in a linear response, making interpretation difficult. On the contrary, As exhibits the opposite behaviour. Thus, in the presence of Phs (both Ph and Ph-TM conditions), the As concentration in the interstitial water is significantly higher, regardless of its decreasing temporal trend observed in all conditions (discussed below). A competitive interaction between Phs and arsenate for sorption sites in the soil at alkaline pHs, resulting in the desorption of As, could explain this pattern. Marzi et al. [102] investigated the effects of various competitive agents (including phosphate, citrate, oxalate, humic acid, and fulvic acid) on the adsorption of As in calcareous soils. Results revealed that phosphate, citrate, and oxalate negatively affected the adsorption of As due to the competition for soil sorption sites. There are several studies that describe the competitive interaction between As and drugs (e.g., CIT, ATE) for binding sites, but all of them consider the human body, and there is a paucity of information about these interactions in the context of environmental research [24,32].

The temporal trend of TM concentrations over the 45 days of irrigation is shown in Figure 5b. Overall, three patterns are observed: (i) concentrations decrease in all investigated conditions (cases of Cu, As, Ni, Pb, and Cd), (ii) the concentration increase in all investigated conditions (case of Zn); and (iii) the concentration exhibits a mixed behaviour (case of Cr). In calcareous soils, certain metal retention mechanisms such as diffusion in the crystal defects (e.g., Cd), the formation of stable associations with humic substance (e.g., Cu), and the nucleation of microprecipitates on the calcite surface (e.g., Pb) occur slowly (ageing effect) and are responsible for the depletion of these TMs from the interstitial water [39]. Competitive binding for sorption sites of these TMs with a high affinity for soil colloids may be responsible for the increase in Zn in interstitial water, whose desorption over time would be favoured. In calcareous soils, arsenate quickly sorbs onto calcite but since it is not readily incorporated into the calcite crystal lattice, desorption of arsenate from calcite is fast and complete within hours [103]. Then, as reported by Marzi et al. [104], ageing significantly increases As sorption onto soils and leads to a more intense irreversibility of its adsorption (hysteresis). This behaviour agrees with the high As concentrations detected in the interstitial water in week 1 and its subsequent gradual decrease over time. Although the As concentration in the interstitial water decreases over time, it always maintains a higher level in Ph and Ph-TM than in C and TM conditions, as mentioned above. The occurrence of competitive reactive processes among TMs within the ageing, as well as the presence of Phs, affects the metallic composition of the interstitial water. More specifically, in C and TM conditions, Zn, Cu, As, and Ni appear in similar concentrations in the interstitial water in week 1 (around 14 µg L^−1^), while Zn is the major element in week 6 (~30 µg L^−1^). In contrast, under Ph and Ph-TM conditions, As is the major element in week 1 (~20 µg L^−1^), with Zn, Cu, and Ni concentrations being around 11 µg L^−1^. In week 6, the As concentration notably decreases to 13 µg L^−1^, and Zn is again the major element (~23 µg L^−1^). In the case of Cr, the concentration increases over time in C and TM conditions, but it remains constant in Ph and Ph-TM ones. As mentioned above in this section, the formation of drug–Cr complexes has been widely described in clinical research. The formation of surface complexes between Cr and Phs may be responsible for enhancing sorption processes and, therefore, continuously depleting Cr from the interstitial water. In the absence of drugs, Cr concentration in the interstitial water increases over time. Chromium is 100% present as a monovalent hydroxyl species and could be readily desorbed by competitive interaction with other TMs with a greater affinity for soil colloids. Indeed, the low Cr adsorption rate onto soils with high ECC content and soil pH has been recently described [105].

#### 3.2.2. TM Accumulation in the Soil

**Figure 6:**
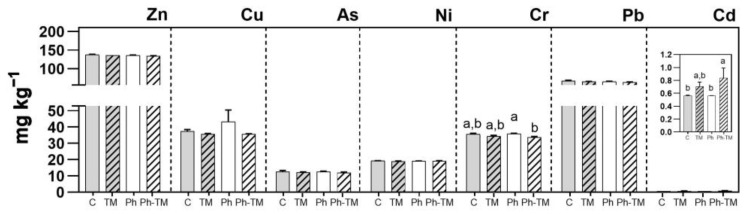
Total average content of trace metals and metalloid (dry weight) in soils from C, TM, Ph, and Ph-TM conditions after the 45-day irrigation period. Error bars are standard deviation. For comparative purposes, TMs appear in order of decreasing concentrations in the interstitial water. Different letters indicate significant differences among conditions at *p* < 0.05 after one-way analysis of variance. In cases where there are no significant differences, nothing is indicated.

The seven selected TMs are detected in the soil after the 45-day irrigation period. Figure 6 shows the concentrations of TMs under C, TM, Ph, and Ph-TM conditions. Zinc, Pb, and Cu occur with the highest content. The average TM content in the soil from the highest to the lowest is as follows: Zn (136 mg kg^−1^) > Pb (65.6 mg kg^−1^) > Cu (38.1 mg kg^−1^) > Cr (34.9 mg kg^−1^) > Ni (19.1 mg kg^−1^) > As (12.4 mg kg^−1^) > Cd (0.7 mg kg^−1^). The TM content detected agrees with that usually reported for agricultural soils in the Mediterranean area [101]. Zinc, Pb, Cu, Cr, and Cd are detected with contents higher than the 90th percentile reference values (RV90) in the soils of Madrid (Order 2770/2006, [106]; RV90 = 73 mg kg^−1^ Zn, 30 mg kg^−1^ Pb, 20 mg kg^−1^ Cu, 32 mg kg^−1^ Cr, and 0.22 mg kg^−1^ Cd). The soil has a background level of metallic contamination since it has been constantly irrigated over the course of years with surface water containing TMs. Additionally, as part of the agricultural practices developed in the area, a variety of herbicides, insecticides, and fungicides, which usually contribute to the presence of metals such as Cu, Zn, and As in the soil [100], have been periodically applied. Nonetheless, the Reference Generic Values (RGVs) for contaminated soils reported in Order 2770/2006 [106] and the maximum levels established by the European Union (Directive 86/278/EEC, n.d.) [107] are not exceeded.

The TM content in the soil is not significantly higher in TM-spiked conditions (TM and Ph-TM) than in unspiked ones (C and Ph), despite us having spiked the irrigation water with a TM concentration 1 or 2 orders of magnitude higher than that occurring under real conditions at the field site. In fact, the TM contents in the soil remain at the levels observed in the field prior to the bioassay [3]. Such behaviour is probably related to the fact that the TM-spiked concentrations are very low when compared to TM contents that have been accumulating in the soil over the course of years (µg L^−1^ vs. mg kg^−1^). Indeed, a calculated total TM content that potentially can accumulate in the soil after 45 days of irrigation (considering the spiked TM concentration, the irrigation volume throughout the bioassay, and the weight of the soil per pot) indicates a content of 0.21 mg kg^−1^, which represents less than 2% of the TM content already present in the soil. This would explain the difficulty of detecting quantifiable differences between conditions and why the pattern observed in the interstitial water with Cu, Cr, and As is not observed in this matrix. The only exception is represented by Cd, as shown in Figure 6. Under the Ph-TM condition, the total content of Cd is significantly higher (about 30%) than that in the C condition. Nevertheless, this result should be taken with caution since the content of Cd in the soil is very low (0.56 ± 0.01 mg kg^−1^ and 0.83 ± 0.09 mg kg^−1^ for C and Ph-TM, respectively). The fact that there is no significant increase in the Cd content in Ph-TM with respect to the TM condition makes us assume that the presence of Phs could have an effect on Cd accumulation but that it should not be very important. Instead, it can be concluded that almost all the Cd is adsorbed onto soil in TM-spiked conditions, in the presence or absence of drugs. Cadmium data reported for the interstitial water (<0.06% of Cd remain in solution) support this hypothesis.

#### 3.2.3. TM Uptake and Translocation by Lettuce

**Figure 7:**
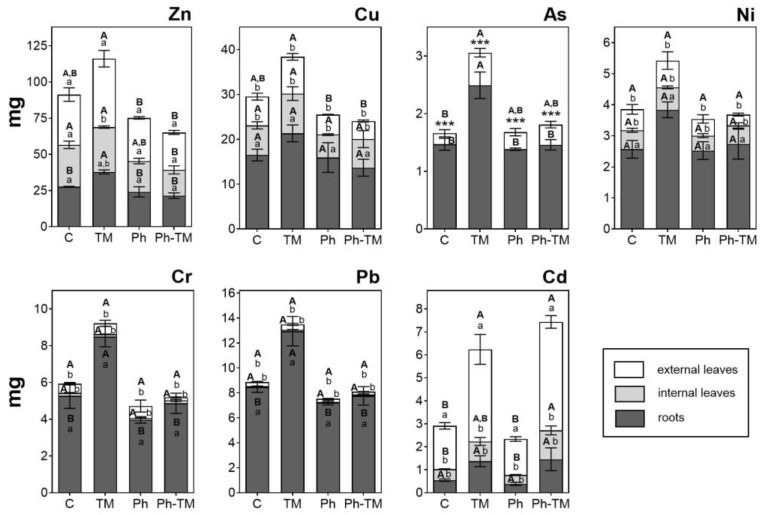
Total average amount of trace metals and metalloid in external leaves, internal leaves, and roots of lettuce plants grown in C, TM, Ph, and Ph-TM conditions after the 45-day irrigation. Error bars are standard deviation. For comparative purposes, trace metals and metalloid appear in order of decreasing concentrations in the interstitial water. Different letters indicate significant differences, among conditions for each tissue (uppercase) or among tissues for each condition (lowercase), at *p* < 0.05 after one-way analysis of variance. The amount of As in internal leaves was lower than the LOQ; thus, significant differences between external leaves and roots for each condition were calculated by a Student’s *t*-test. *** indicates significant differences at *p* < 0.001.

The seven selected TMs are detected in the lettuce tissues after the 45-day irrigation period. Figure 7 shows the total amount of TMs in external leaves, internal leaves, and roots under C, TM, Ph, and Ph-TM conditions. In general, a greater uptake of TMs is observed under the TM condition. Zinc, followed by Cu, is present with the highest amount, and these results agree with those previously reported for lettuce bioassays carried out with agricultural soils of the Mediterranean area [108,109]. The average TM amount taken up by plants (sum of leaves and roots) under the TM condition from the highest to the lowest is as follows: 116.1 mg Zn > 38.8 mg Cu > 13.5 mg Pb > 9.2 mg Cr > 6.2 mg Cd > 5.4 mg Ni > and 3.1 mg As. This sequence is similar to that obtained in the soil, where the content of Zn, Cu, Pb, and Cr predominates over the rest of the TMs. On the contrary, it does not correspond to what is observed in the interstitial water, where Cr and Pb are detected with very low concentrations. As concluded for drugs, the uptake of TMs seems to be more related to the soil accumulation pattern. Bioavailability is the result of the root–soil–microorganism interaction. Plant exudates and microbial metabolites in the rhizosphere environment may promote the transport of TMs into the plants, increasing the bioavailability of those TMs sorbed onto the soil. Thus, roots extrude protons to acidify the rhizosphere (releasing TMs from hydroxides) and to displace divalent metal ions from exchangeable positions, in addition to creating a large membrane potential that is a key driving force for cation uptake [110]. In such a context, carbonate, organic, and fine mineral fractions play a key role in governing TMs’ bioavailability to lettuce in calcareous soils [108].

Differentiating by tissues, Zn is the only TM that proportionally occurs in the aerial and underground parts (average values are 47% in roots, 27% in internal leaves, 31% in external leaves). Copper, As, Ni, Cr, and Pb mainly accumulate in the roots (average values are 67%, 90%, 79%, 93%, and 97%, respectively), and Cd in external leaves (average value 72%). The TFs calculated for Cu, As, Ni, Cr, and Pb range from 0.03 to 0.6 (TF < 1 in all cases), revealing the low tendency of TMs to translocate towards the aerial tissues of lettuce plants. Similar results were already described in the literature [111]. In contrast, Zn and Cd TFs range from 1.2 to 1.5 and from 2.4 to 3.3, respectively, indicating their greater translocation potential. In multi-elemental mixtures, TMs compete for binding to the root surface prior to their uptake. In such a context, Cd and Zn could bind more easily to transport sites in the root cell membrane, where they are subsequently taken up and translocated, than other TMs that bind more strongly to soil colloids or even precipitate on the root surface (e.g., Cu and Pb) [108]. Cadmium is not an essential element for plants, and it is probably taken up by transporters for essential nutrients, via Ca^2+^, Fe^2+^, Mn^2+^, and Zn^2+^ uptake systems [110], therefore explaining its greater amount in the aerial parts of lettuce plants.

The Ph-TM mixture spiked in the irrigation water has a significant negative effect on the amount of bioaccumulated TMs. Thus, the amount of several TMs significantly decreases in the Ph-TM condition with respect to the TM condition as follows: 41% As, 43% Cr, 40% Pb in roots, 51% Cu in external leaves, and 44% Zn in all tissues. The results suggest that TM uptake and/or translocation is hampered by the presence of Phs, probably due to the above-discussed Ph-TM complexes. This pattern has been described as being highly dose-dependent. Thus, co-contamination of low concentrations of Cu and tetracyclines may exert antagonistic effects on their uptake by plants [22]. At high concentrations, metal sorption into the root surface may occur to alleviate the adverse effects of the contamination. Conversely, the amount of Cd in lettuce tissues is not significantly different in the presence of Phs, although a tendency to be higher in the Ph-TM condition is observed. Up to three times more Cd has been detected in lettuce tissues in TM-spiked pots (TM and Ph-TM) than in unspiked ones (C and Ph), which is in agreement to what is observed in the soil. This result suggests that the uptake and translocation of Cd is directly related to the amount of Cd present in the soil and that the presence of drugs has a minor effect. According to these results, the BCF_soil_ of Zn, Cu, As, Ni, Cr, and Pb is <1 and ranges from 0.2 (Zn, As, Ni, Cr, and Pb) to 0.5 (Cu) under the TM condition, denoting a low tendency to be taken up by lettuce. For these TMs, BCF_soil_ values are significantly greater when TMs are spiked in the absence of Phs (TM condition). In the case of Cd, the BCF_soil_ value is the highest in the Ph-TM-spiked pots (3.3), followed by the TM pots (1.5), and is the lowest in the C and Ph pots (1.0).

### 3.3. Impact on Lettuce Growth and Nutrient Balance

**Figure 8:**
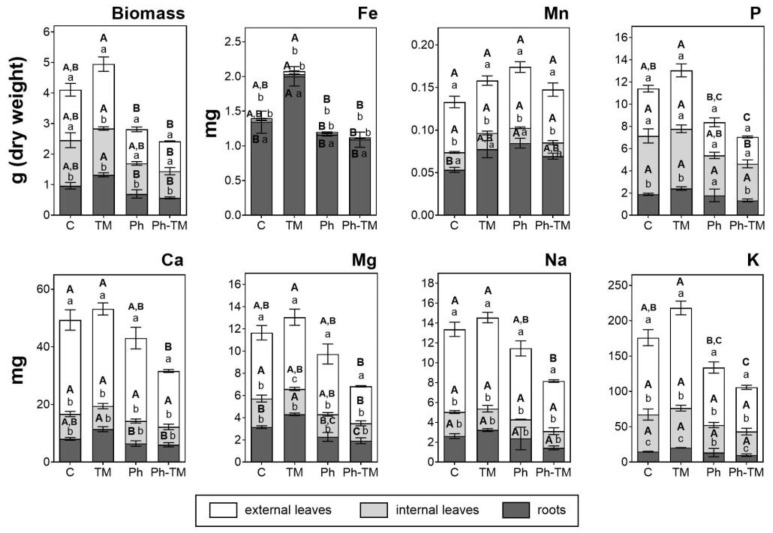
External leaf, internal leaf, and root biomass (dry weight) and total average amount of major elements in external leaves, internal leaves, and roots of lettuce plants grown in C, TM, Ph, and Ph-TM conditions after the 45-day irrigation. Error bars are standard deviation. Different letters indicate significant differences, among conditions for each tissue (uppercase) or among tissues for each condition (lowercase), at *p* < 0.05 after one-way analysis of variance.

Overall, the presence of Phs has a negative impact on lettuce growth and nutrient uptake, and the interaction of Phs and TMs causes an imbalance in nutrients, whereas the exclusive presence of TMs favours the growth of the plants (Figure 8). Total biomass decreases by 30% (Ph) and 40% (Ph-TM) with respect to unspiked soils (C, TM). This decrease was significant when comparing Ph- and Ph-TM-spiked pots with respect to TM-spiked ones. The calculated tolerance indexes of the lettuce grown in Ph-TM-spiked pots are lower than those grown with only Phs or TMs. However, the application of the ANOVA test reveals that this decrease is not statistically significant. The obtained results are similar when calculations are done by differentiating among plant tissues. Other parameters, such as the number of leaves, height, and diameter do not exhibit significant differences among conditions. Different impacts of drugs on crops have been described in the literature so far. For example, Papaioannou et al. [16,17] reported the lack of any effects in the yield of common beet (*Beta vulgaris* L.) irrigated with treated municipal wastewater containing a range of drugs (including SUL, TRI, CBZ, and CLA, among others). However, the authors observed that the beet yield increased with the increase in the caffeine and salicylic acid contents in the soil. In contrast, Carvalho et al. [112] concluded that drugs can induce phytotoxicity in plant crops and that the specific effects will depend on the drug, its concentration, and the plant species. The variability in reported results may be related to the wide range of investigated drug concentrations (from μg L^−1^ to mg L^−1^) and evaluated parameters (root length, germination, pigments, etc.), sometimes so great that comparisons are anything but straightforward. To the authors’ knowledge, the impact of Phs on plant growth has not been accurately elucidated, and it is still not clear if the potential negative effect is due to the direct damage of the plants or of their symbiotic microorganisms [113]. It is interesting to note that under certain conditions (e.g., low concentrations of harmful drugs), plants are capable of implementing a series of biochemical mechanisms to cope with drug toxicity and eliminate harmful substances [112].

Differentiating by tissue, lettuce biomass (DW) in TM-spiked pots significantly increases with respect to unspiked pots (C) as follows: 28%, 2%, and 37% for external leaves, internal leaves, and roots, respectively. Lettuce growth stimulation at low concentrations of TMs has been previously reported in the literature, and it is attributed to the increased level of oxidative stress, which may cause an increase in reactive oxygen species (ROS) signalling [114]. Further, trace amounts of some TMs, including Cu, Zn, and Cr, are essential to plant metabolism [115]. Therefore, it can not be ruled out that the concentrations of TMs used in this bioassay are below the levels that are toxic to lettuce, or they are not in bioavailable form. However, in the presence of drugs (with and without TMs), the biomass of external leaves and roots decreases significantly. In the internal leaves, the same trend is observed, but is significant only in Ph-TM vs. TM pots. The results show that the biomass stimulation produced by TMs is prevented in the presence of drugs. The lower uptake of TM (except in the case of Cd) in the Ph-TM condition may have been influenced by this decreased biomass. To evaluate whether some functions have been altered, the total amount of major elements in lettuce tissues was determined (Figure 8). In all cases, K is the element absorbed and translocated in the greatest quantity, followed by Ca, Na, and Mg. On the contrary, Fe and Mn are the major elements with the lowest amounts in the plant tissues. Overall, Fe accumulates in roots, Mn in external leaves and roots, while Ca, Mg, Na, K, and P translocate towards the aerial parts (mainly external leaves). As occurs for plant growth, the nutrient content in plant tissues increases in the presence of TMs. This pattern agrees with a previous study evaluating the impact of TMs in different varieties of lettuce, and it was attributed to plant protective strategies to resist the toxic effects of TMs [108]. In contrast, our results show that major element uptake and/or translocation is hampered by the presence of Phs, mainly in the Ph-TM condition. Overall, the amounts of most major elements significantly decreases in Ph-TM-spiked pots with respect to TM-spiked ones: Na, K (external leaves), Fe, Ca, Mg (external leaves and roots), and P (external and internal leaves). The complexes formed between Phs (or their TPs) with some cations, such as Ca^2+^, might induce the deficiency of such cations in the plants [112]. An imbalance in the uptake of nutrients can affect chlorophyll production and stability, among other essential functions [114]. Plant responses to nutrient deficiency could involve an increased surface area of the root system to optimise nutrient uptake in a localised part of the soil [110]. In our case, results show that neither Phs nor the combination of Phs and TMs led to a significant adverse impact on most of the visible symptoms (height, number of leaves, etc.), except in the case of biomass. However, there is a potential impact on several essential functions of plants, which can be indirectly observed by the imbalance in nutrients. This should be taken into account for future studies in which low concentrations of contaminants similar to those observed in the environment are evaluated. Moreover, long-term nutrient deficiencies in crops due to the presence of these contaminants could also have an impact on human health, due to the development of diseases such as iron deficiency anaemia (IDA), neuro-behavioural problems due to Zn deficiency, etc. [116]. These are known as “hidden hunger”, and they are a form of malnutrition that occurs when the intake of minerals and vitamins (particularly Fe, Zn, iodine, and vitamin A) are insufficient to maintain good health and development [117]. In a similar way, Christou et al. [118] found that tomato productivity was not affected by the presence of Phs (DIC, SUL, TRI), but it induced an impact on the fruit quality attributes.

### 3.4. Health Risk Assessment

The Phs detected in the lettuce leaves are assigned to Class III, a category that includes substances for which a strong initial presumption of safety is not allowed or whose structural properties suggest significant toxicity [61,62]. Exceptions are represented by the anti-inflammatory IBU that belongs to Class I (low order of oral toxicity), VEN along with its TP OVEN, which are Class II compounds (intermediate order of oral toxicity), and the TP CBZPOX, which is considered a compound having potential genotoxicity (TTC: 0.0025 μg kg^−1^ day^−1^). Under both Ph and Ph-TM conditions, calculated DCs to reach TTC values for adults, children, and toddlers are far below (from 3 to 9 orders of magnitude) the DI for each age range. Therefore, independently of the experimental conditions, the contents of Phs in the lettuce’s edible parts indicate an insignificant threat to human health. Due to the genotoxicity recognized by its molecular structure, CBZPOX is the only drug with values of DC closer to the DI of lettuce for all three age ranges. For this compound and under Ph conditions, TTC could be achieved in adults, children, and toddlers with a consumption of 120 g, 41 g, and 20 g, respectively. However, in the presence of TMs, these values decrease to 90 g, 31 g, and 15 g as a consequence of a greater average concentration of CBZPOX in the lettuce leaves. However, calculated values are still below the DI reported for lettuce by the FAO and WHO. When applying the HQ approach to Ph contents in the lettuce leaves, the predicted risk is highly negligible for all the investigated Phs under both spiked and unspiked conditions. Indeed, the HQ score is always well below the threshold value of 1, and the HI, calculated as the sum of HQs, was only 0.06 and 0.08 under the Ph and Ph-TM conditions, respectively. The increase in the HI when TMs are together with Phs agrees with the highest contents in lettuce leaves observed for certain Phs under the mentioned experimental conditions.

The HQ values of TMs are <1 in all cases. Overall, the HQ of Cd is the highest (ranging from 0.02 to 0.19), followed by As (ranging from 0.0027 to 0.014), Zn (ranging from 0.0005 to 0.0017), and Pb, Ni, Cu, and Cr < 0.001. The interaction of Phs and TMs impacts the HQ values in certain TMs. Thus, the HQ value of Cd increases from 0.08 to 0.18, in TM and Ph-TM, respectively. A slight increase is also observed in the case of Pb (1.2 times) and As (1.1 times). Similar results are obtained by differentiating by children. The HQ values for toddlers are lower, although they showed the same pattern: from 0.02 to 0.06 (Cd), from 0.0027 to 0.0047 (Zn), and <0.001 (rest of TMs). The HIs for each age range and condition is lower than 1 in all cases: 0.1 and 0.2 (adult), 0.1 and 0.21 (child), and 0.03 and 0.07 (toddler), in TM and Ph-TM, respectively. The concentrations of TMs used in the present work is low in order to be environmentally relevant. Therefore, it is to be hoped that they do not pose a high risk to health. Although there would be no relevant risk for consumers, considering the HQ approach, this would be greater in the case of lettuces irrigated with water with TMs in the presence of Phs.

Cadmium and Pb are included in the list of contaminants in leafy vegetable foodstuffs. The levels of Cd in leaves expressed on a wet-weight basis are above the European Union limits established for leafy vegetables (0.1 mg kg^−1^) in TM-spiked pots (0.12 and 0.27 mg kg^−1^ in TM and Ph-TM, respectively) and very close to the limit in TM-unspiked pots (0.07 and 0.08 mg kg^−1^ in C and Ph, respectively) (EC 1881/2006, [119]). So, results show that Cd levels exceed 2.7 times the maximum limits in the presence of the Phs. Staple foods constitute between 40 and 60% of the total dietary intake of Cd, so attention should be paid to leafy salad vegetables, such as lettuce and spinach, due to their hyperaccumulating capacity of Cd [120]. Although Cd levels are apparently low, they should not negligible. Chronic intake of low-level dietary Cd is associated with an increased risk of developing obesity, cardiovascular disease, diabetes, and metabolic syndrome. Cadmium absorption increases during pregnancy, and it can enter the placenta, potentially affecting placental development and function, with adverse long-term health implications [11]. In the case of Pb, the established limit levels are never exceeded (0.1 mg kg^−1^) since the calculated values are one order of magnitude below the limit.

### 3.5. Impact on Soil Health

**Figure 9:**
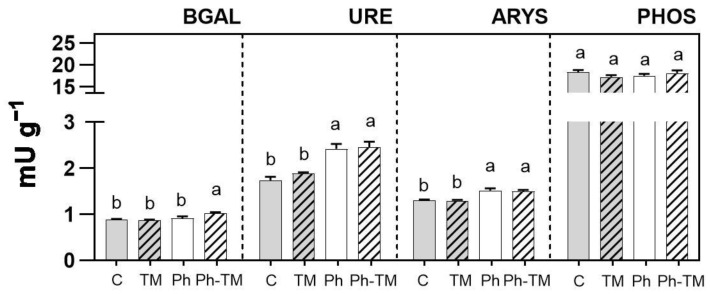
β-Galactosidase (BGAL), urease (URE), aryl sulfatase (ARYS), and phosphatase (PHOS) activity average values in soils from C, TM, Ph, and Ph-TM conditions after the 45-day irrigation. Error bars are standard deviation. Different letters indicate significant differences among conditions at *p* < 0.05 after one-way analysis of variance.

The presence of Phs in the irrigation water impacts on three of the four enzyme activities, as shown in Figure 9. Overall, enzyme activity after 45 days of irrigation is not inhibited, but stimulated or not affected under pharmaceutical stress. The presence of TPs (ATEC, CBZPOX, and COT) in the soil (Section 3.1.2) partially originates from the biochemical activity of soil microorganisms, confirming its stimulation in the presence of drugs. Overall, three different patterns are observed: (i) a significant increase in enzyme activity under Ph-TM conditions (case of BGAL), (ii) a significant increase in enzyme activity in the presence of Phs with and without TMs (cases of URE and ARYS), and (iii) no significant changes in enzyme activity under Ph and/or TM conditions (case of PHOS).

A wide variability in results has been found in the literature regarding the impact of drugs on soil enzyme activity, which is highly dependent on soil type, pharmaceutical compound, dose, and exposure time. Four microbial responses to Ph amendment (SUL, CIT, clindamycin, fexofenadine, and irbesartan) were identified by Frková et al. [121] at different contact times (from 1 to 61 days) in a set of European agricultural soils: stimulation, inhibition, stress, and/or dormancy. In the short term, they reported a shift in the microbial community, reflected by changes in the G+/G− and bacterial-to-fungal (B/F) ratios, in particular in response to SUL and a Ph mixture, but this was shortly replaced by stimulation, suggesting a high resilience of the microbial community. In the same way, a short-term inhibition enzyme activity (URE and PHOS) has been reported in the first days of contact with different Phs, and then an increase, with a net balance of either inhibition (sulfamethazine or enrofloxacin) [38,40] or, in contrast, stimulation (DIC, IBU, naproxen, or ketoprofen) [122]. In the present study, the temporal trend of enzyme activities over 45 days of irrigation has not been evaluated, so an initial inhibition can not be ruled out. After 45 days, the stimulation observed of the enzyme activity in the presence of drugs is consistent with a selective pressure of certain populations. This corresponds to the fact that at the field site, the soil used in the bioassays has been irrigated for years with water impacted by a mixture of TMs and Phs, including antibiotics, most of them spiked in this study (i.e., SUL, TRI, CLA, LIN, and METRO). The initial inhibition reported by Yang et al. [38,40] could be attributed to the fact that the Phs of the study were antibiotics. In this sense, Liu et al. [123] evaluated the impact of six antibiotics (SUL, TRI, sulfamethazine, chlortetracycline, tetracycline, and tylosin) individually spiked in a range of concentrations (1–300 mg kg^−1^) and observed that soil PHOS activity was inhibited during the 22 days of incubation. However, other authors did not observe a visible inhibition effect of the antibiotics SUL, ofloxacin, and roxithromycin and the antiepileptic CBZ on constructed wetland substrate enzyme activities (URE, dehydrogenase, and catalase) [124].

A Ph-TM synergistic interaction is observed in the case of BGAL, which is significantly stimulated. Some authors have observed synergistic effects between TMs and Phs, but with results opposite to ours. For example, the combined contamination of Cu and sulfamethazine [40] or Cu and enrofloxacin [38] showed a greater inhibitory effect on soil URE and PHOS activities than individual applications. The authors attributed these results to the increase in the bioavailability and/or toxicity of TMs in the presence of Phs or to changes in the kinetics of interaction with microorganisms and/or enzymes. This is exactly the opposite of what was reported in the present study, where the Ph-TM combination, in general terms, decreased Ph and TM bioavailability for plants or their mobility in soils. The impact on enzyme activity reflects an alteration in the microbial population which, in turn, could also account for the different uptake by lettuce plants observed of some target contaminants in different Ph-TM pots, since bioavailability is the result of the root–soil–microorganism interaction. Also, as mentioned in Section 3.3, it is not clear if the impact of contaminants on plant growth is due to the damage to plants or to their symbiotic microorganisms [113]. Kong et al. [21] also reported an additive synergistic effect of oxytetracycline and Cu on the functional diversity of soil microbial communities, which they attributed to the sensitivity of bacteria degrading polymers and carbohydrates towards oxytetracycline and bacteria degrading carboxylic acids, polymers, and amines towards Cu. Because each contaminant separately impacted these populations in the same way, the authors ruled out the possibility that potential Ph-TM complexes reduced the toxicity and/or availability of the contaminants, contrary to what was attributed in the present study and discussed in previous sections.

PHOS activity does not show significant differences among the conditions, as was also observed by Ma et al. [23] when they studyied the effect of dose (0.03 to 30 mg kg^−1^) and application frequencies of oxytetracycline in an agricultural soil on the activities of soil microorganisms and enzymes. In contrast, Cycon et al. [122] reported an increase in PHOS activity in the presence of NSAID drugs (e.g., DIC, IBU), attributed to the stimulation of indigenous fungi, whose number significantly increased over the experimental period. Fungi are known to be soil producers of PHOS and NSAID-degrading microorganisms. Cycon et al. [122] added between two and three orders of magnitude higher drug concentrations than the present study. In TM-spiked soil samples, de Santiago-Martín et al. [125] observed that PHOS activity was the least inhibited, attributing such a behaviour to a lesser sensitivity of this enzyme, or of the organisms that produce it, to target TMs, and/or to a favoured persistence of total and immobilised PHOS activity in soil colloids such as OM.

The presence of TMs does not exert an inhibitory effect, in contrast to what was previously observed in similar calcareous agricultural soils of the Mediterranean area [125]. The spiked TM concentration (100 µg L^−1^) does not seem to be high enough to affect the studied enzyme activities, despite being up to two orders of magnitude higher than that detected in the irrigation water in the field. This result was attributed to the fact that this soil already presents a background level of TMs, with an adapted microbial community. This could have favoured the selection of metal-resistant microorganisms, releasing enzymes poorly affected by the presence of TMs in the water–soil interface. The inhibition of some soil enzyme activities in the presence of TMs can decrease over time due to (i) the adaptation of soil microorganisms to the polluted environment, and (ii) the ageing effect that makes TMs less available to microorganisms [126]. This ageing effect of TMs in the soils has been detailed in Section 3.2.1. Besides, de Santiago-Martín et al. [125] reported that in those calcareous soils that had a higher fine-size mineral fraction and OM content, the inhibition of microbial biomass, as well as exocellular hydrolytic enzymes released into the medium, was considerably lower. The authors attributed this to several factors, which probably occur simultaneously: (i) enzyme protection in humus–hydrolase and/or clay–humus–hydrolase complexes against proteases, (ii) physical stabilisation of microorganisms producing enzymes, and (iii) metallic sorption reducing its availability. In the present work, the content of OM is moderate (2%) but the silt content is high (62%). The fine silt fraction has been identified as being a key mineral fraction in the stabilisation of certain exocellular enzymes, such as ARYS [127], preventing its microbial or chemical degradation.

## 4. Conclusions

This study proves that the co-occurrence of Phs and TMs has an impact on the water–soil–plant continuum. Depending on the Ph, the TM, and the environmental compartment, this impact is synergistic, antagonistic, or without significant effect. In this sense, the most relevant results obtained by our research can be summarised as follows:

- At the soil–water interface, the levels of occurrence of Phs in the interstitial water is mostly attributed to their properties. Indeed, neutral and anionic drugs with a hydrophilic nature (VAL, SUL, IBU, GEM, CBZ, and METRO) are detected in this matrix with the highest concentrations. An over-time accumulation trend is observed for certain Phs (CBZ, CBZPOX, LORZ, and DIA), and such behaviour may be a matter of concern. TMs do not have an impact on Ph concentrations. In contrast, the presence of Phs exerts an antagonistic effect on Cu and Cr concentration, attributed to the increase in their sorption by the formation of surface complexes and/or cation bridges. At the same time, Phs have a synergistic effect on As concentration, probably due to competitive interactions for sorption sites.
- In the soil, the highest contents of drugs were attributed either to their properties (positively charged, e.g., VEN and FLE) or to their high persistence to degradation (CBZ). The contents of several Phs were higher under the Ph-TM condition; however, only LIN, TRI, and DIA show a significant increase. On the other hand, the TM contents in the soil are not significantly affected, neither by the presence of Phs (exceptions are observed exclusively for Cr and Cd), nor by the spiked TMs. Such behaviour is probably related to the fact that TM-spiked concentrations are very low when compared to the background TM contents resulting from the accumulation over years from agricultural practices in the calcareous soils.
- The amounts of both Phs and TMs taken up by lettuce plants correlate with their contents in the soil, suggesting that desorption from soil colloids is not a limiting factor for their bioavailability, and thus, for plant uptake. Anionic Phs tend to remain in roots (SUL, IBU, and GEM), while neutral, zwitterionic, and cationic Phs are found in leaves. Considering external lettuce leaves, the co-occurrence of Phs and TMs exerts both antagonistic and synergistic effects on CBZ and DIA, respectively. Moreover, the metabolism of CBZ seems to increase in the plant in the presence of Phs and TMs. Overall, under the Ph-TM condition, the BCF_soil_ of Phs decreases and TF increases or decreases depending on the drug. In the case of TMs, with the exception of Cd, the presence of drugs exerts an antagonistic effect, negatively affecting their uptake and translocation. The uptake of Cd towards the lettuce leaves is slightly favoured under the Phs and TMs’ co-occurrence.
- Results of the health risk assessment indicate that the contents of Phs in the lettuce edible parts do not pose a threat to human health, independently of tested conditions. However, in the presence of the Phs, Cd levels exceed by 2.7 times the maximum limits set for leafy vegetable foodstuffs. This result may be a matter of great concern, since a chronic intake of Cd, even at a low level, can have direct consequences on health, mainly in vulnerable stages such as pregnancy and childhood.
- Crop quality is negatively affected when Phs and TMs co-occur. The impact is observed in the decrease in biomass production and in the nutrient imbalance. Results show that major element uptake and/or translocation is hampered, possibly leading to nutrient deficiencies in crops that in the long term cause a “hidden impact” on human health.
- Soil enzyme activity is rather stimulated under pharmaceutical stress. This is consistent with selective pressure in certain populations. The co-occurrence of Phs and TMs favours BGAL activity, while ARYS and URE are stimulated in the presence of Phs with or without TM spiking. The impact on enzyme activity reflects an alteration in the microbial population, which, in turn, may account for the different uptake by lettuce plants and degradation rates observed of some target contaminants. Indeed, the degradation of ATE into ATEAC is enhanced under the co-occurrence of Ph and TMs in both interstitial water and soil, likely as a consequence of the stimulation of certain microbial under these conditions. Nevertheless, it is considered that a more detailed analysis of the soil microbial community could provide further insight into how Phs and TMs affect long-term soil health and fertility.

## Figures and Tables

**Table 1:** Pharmaceuticals and transformation products selected in this study.

Compound	CAS Number	ATC Code	Molecular Mass (Da)	Charge State at pH 8.7 ^a^	pKa ^b^	logKow ^b^	logDow ^b^
**Cardiovascular system**							
Flecainide	54143-55-4	C01BC04	414.14	cationic	13.68 (A); 9.62 (B)	3.19	2.22
Atenolol	29122-68-7	C07AB03	266.16	cationic	14.08 (A); 9.27 (B)	0.43	−0.25
*Atenololic acid*	56392-14-4	-	267.15	zwitterionic	3.54 (A); 9.27 (B)	−1.24	−1.33
Propranolol	525-66-6	C07AA05	259.16	cationic	14.09 (A); 9.27 (B)	2.58	1.91
Enalapril **	75847-73-3	C09AA02	376.19	anionic	3.88 (A); 5.21 (B)	0.53	−1.27
Valsartan	137862-53-4	C09CA03	435.53	anionic	4.41 (A); 3.35 (B)	5.00	0.22
Gemfibrozil	25812-30-0	C10AB04	250.16	anionic	4.42 (A)	4.39	0.93
**Anti-infectives for systemic use**							
Trimethoprim	738-70-5	J01EA01	290.14	neutral	7.16 (A)	1.28	1.27
Sulfamethoxazole	723-46-6	J01EC01	253.05	anionic	5.86 (A); 1.97 (B)	0.79	−0.15
*N4-acetylsulfamethoxazole*	21312-10-7	-	295.06	anionic	5.58 (A); 0.38 (B)	0.86	−0.08
Clarithromycin *	81103-11-9	J01FA09	747.48	cationic	12.46 (A); 9.00 (B)	3.24	2.76
Lincomycin	154-21-2	J01FF02	406.21	neutral	12.37 (A); 7.97 (B)	−0.32	−0.39
Metronidazole	443-48-1	J01XD01	171.06	neutral	15.41 (A); 3.03 (B)	−0.46	−0.46
**Musculo-skeletal system**							
Ibuprofen **	15687-27-1	M01AE01	206.13	anionic	4.85 (A)	3.85	0.48
Diclofenac *	15307-86-5	M01AB05	296.02	anionic	4.01 (A); −1.08 (B)	4.26	0.76
*4-Hidroxi-diclofenaco*	64118-84-9	-	311.01	anionic	3.77 (A); 0.41 (B)	3.95	0.09
**Nervous system**							
Codeine	76-57-3	N02AJ09	299.15	cationic	13.78 (A); 8.89 (B)	1.34	0.3989
Metamizole (antipyrine)	5907-38-0	N02BB02	351.09	anionic	−1.42 (A); −0.54 (B)	−0.82	−2.25
*4-Aminoantipyrine (AA)*	83-07-8	-	203.11	neutral	0.07 (A)	0.34	0.34
*4-Dimethilaminoantipyrine (DAA)*	58-15-1	-	231.14	neutral	3.66 (B)	1.15	1.15
*4-Formylaminoantipyrine (FAA)*	1672-58-8	-	231.10	neutral	9.86 (A); 0.18(B)	0.11	0.08
Acetaminophen (paracetamol) **	103-90-2	N02BE01	151.06	neutral	9.46 (A)	0.91	0.84
*3-Metoxy-acetaminophen*	3251-55-6	-	181.07	neutral	9.92 (A)	0.75	0.72
Carbamazepine	298-46-4	N03AF01	236.09	neutral	15.96 (A)	2.77	2.77
*Carbamazepine epoxide*	36507-30-9	-	252.09	neutral	15.96 (A)	1.97	1.97
Diazepam	439-14-5	N05BA01	284.07	neutral	13.04 (A); 2.92 (B)	3.08	3.08
Lorazepam **	846-49-1	N05BA06	320.01	neutral	10.61 (A)	3.53	3.52
Citalopram	59729-33-8	N06AB04	324.16	cationic	10.38 (B)	3.76	2
Venlafaxine	93413-69-5	N06AX16	277.20	cationic	14.42 (A); 9.01 (B)	2.74	2.26
*o-Desmethylvenlafaxine*	93413-62-8	-	263.18	cationic	9.80 (A); 8.90 (B)	2.19	2.06
Nicotine †	54-11-5	N07BA01	162.11	neutral	8.58 (B)	1.16	0.92
*Cotinine* †	486-56-6	-	176.09	neutral	4.79 (B)	0.21	0.21

* Watchlists [42]; ** National Health System [43] and Spanish Pharmaceutical Observatory [44]; † life-style compounds of drugs of abuse. TPs are in italic. ^a^ Predominant charge state at pH 8.7 (average value from interstitial water). ^b^ Chemicalize (https://chemicalize.com/), accessed on 1 February 2024. A: acidic; B: basic.

## Data Availability

The raw data supporting the conclusions of this article will be made available by the authors on request.

## Supplementary Materials

The following supporting information can be downloaded at https://www.mdpi.com/article/10.3390/toxics12070457/s1, Table S1. Instrumental parameters and chromatographic conditions for pharmaceutical and transformation product analysis by LC-MS/MS. Table S2. Retention times (t_R_), collision energies (CE), precursors and product ions (Q; quantifier, and q; qualifier) selected for the analysis of pharmaceuticals and transformation products (in italics) in Multiple Reaction Mode (MRM). Table S3. Instrumental detection (LODi) and quantification (LOQi) limits for pharmaceuticals and transformation products (in italics). Absolute and relative recoveries (%) and relative standard deviation values (RSD, %) (*n* = 3) for pharmaceuticals and transformation products (in italics) obtained in soil and plant samples (roots and leaves). Table S4. ICP-MS instrumental parameters. Table S5. Instrumental detection and quantification limits (LODi and LOQi) for element analysis in water samples by ICP-MS. Methodological quantification limits (MQL) for element analysis in soil and plant samples by ICP-MS prior to acid digestion. Table S6. Average concentrations of pharmaceutical and transformation products in the interstitial water from CA and TM conditions over the 45-day irrigation period. Table S7. Total average soil contents of pharmaceuticals and transformation products (dry weight) from CA and TM conditions after the 45-day irrigation period. Table S8. Average concentrations of pharmaceuticals and transformation products in external leaves, internal leaves, and roots of lettuce plants grown in CA and TM conditions after the 45-day irrigation period. Table S9. Pearson’s correlation coefficients calculated among pharmaceutical and transformation products’ concentrations in environmental matrices (lettuce, interstitial water, and soil) and compound physico-chemical parameters in Ph condition. Table S10. Pearson’s correlation coefficients calculated among pharmaceutical and transformation products’ concentrations in environmental matrices (lettuce, interstitial water, and soil) and compound physico-chemical parameters in Ph-TM condition.
