# Supplementary material for: Pharmaceutical and Trace Metal Interaction within the Water–Soil–Plant Continuum: Implications for Human and Soil Health
Source: Toxics. 2024 Jun 25;12(7):457. doi: 10.3390/toxics12070457 (PMC11281246; doi:10.3390/toxics12070457)
Supplement: Supplementary file 1 [file toxics-12-00457-s001.zip › toxics-3050882-supplementary.pdf]

toxics

Supplementary Materials

Pharmaceutical and Trace Metal Interaction within the Water–Soil–Plant Continuum: Implications for Human and Soil Health

Lesly Ayala Cabana [1,2,*], Ana de Santiago-Martín [1], Raffaella Meffe [1], Isabel López-Heras [1] and Irene de Bustamante [1,2]

[1] IMDEA Water Institute, Alcalá de Henares, 28805 Madrid, Spain
[2] Department of Geology, Geography and Environment, University of Alcalá, Alcalá de Henares, 28802 Madrid, Spain
* Correspondence: lesly.ayala@imdea.org

Contents

Chemicals and Reagents.

Sample treatment for the extraction of pharmaceuticals and transformation products.

Table S1. Instrumental parameters and chromatographic conditions for pharmaceutical and transformation product analysis by LC-MS/MS.

Table S2. Retention times (t_R), collision energies (CE), precursors and product ions (Q; quantifier, and q; qualifier) selected for the analysis of pharmaceuticals and transformation products (in italics) in Multiple Reaction Mode (MRM).

Table S3. Instrumental detection (LODi) and quantification (LOQi) limits for pharmaceuticals and transformation products (in italics). Absolute and relative recoveries (%) and relative standard deviation values (RSD, %) (n = 3) for pharmaceuticals and transformation products (in italics) obtained in soil and plant samples (roots and leaves).

Table S4. ICP-MS instrumental parameters.

Table S5. Instrumental detection and quantification limits (LODi and LOQi) for element analysis in water samples by ICP-MS. Methodological quantification limits (MQL) for element analysis in soil and plant samples by ICP-MS prior to acid digestion.

Table S6. Average concentrations of pharmaceutical and transformation products in the interstitial water from CA and TM conditions over the 45-day irrigation period.

Table S7. Total average soil contents of pharmaceuticals and transformation products (dry weight) from CA and TM conditions after the 45-day irrigation period.

Table S8. Average concentrations of pharmaceuticals and transformation products in external leaves, internal leaves, and roots of lettuce plants grown in CA and TM conditions after the 45-day irrigation period.

Table S9. Pearson's correlation coefficients calculated among pharmaceutical and transformation products' concentrations in environmental matrices (lettuce, interstitial water, and soil) and compound physico-chemical parameters in Ph condition.

Table S10. Pearson's correlation coefficients calculated among pharmaceutical and transformation products' concentrations in environmental matrices (lettuce, interstitial water, and soil) and compound physico-chemical parameters in Ph-TM condition.

Chemicals and Reagents

LC/MS-grade acetonitrile (MeCN) and methanol (MeOH) were supplied from Scharlau and Carlo Erba (Spain), respectively. Formic acid (CH_2O_2, purity ≥ 98%), ammonium fluoride (NH_4F), and disodium ethylenediamine-tetraacetate dihydrate ($Na_2EDTA \cdot 2H_2O$) were obtained from Merck (Germany). Ammonium hydroxide (32% NH_4OH), phosphoric acid (85% H_3PO_4, Pharmpur), and disodium ethylenediaminetetraacetate ($Na_2EDTA \cdot 2H_2O$) were purchased from Scharlau (Barcelona, Spain). Hydrochloric acid (32–35% HCl) and sodium dihydrogen phosphate dihydrate ($NaH_2PO_4 \cdot 2H_2O$) were supplied by Fisher Scientific (USA) and by Panreac AppliChem (USA), respectively. Ultra-trace-quality nitric acid (65%) and hydrogen peroxide (H_2O_2) were obtained from analytical-grade reagents from Fisher Scientific (USA) and Sigma-Aldrich.

The analytical standards (purity ≥ 98–99%) for the quantitative analyses of Phs and TPs were purchased from Sigma-Aldrich, except for CIT (97.2% purity), which was provided by the Center for Applied Chemistry and Biotechnology of the University of Alcalá (Spain). Isotopically labelled compounds (purity ≥ 95%) were used as internal standards. [$^2H_7$]-atenolol, [$^2H_{10}$]-carbamazepine, [$^2H_6$]-citalopram, [^{13}C,$^2H_3$]-clarithromycin, [$^2H_3$]-codeine, [$^2H_3$]-ibuprofen, [$^2H_4$]-nicotine, [$^2H_7$]-propranolol, [$^{13}C_6$]-sulfamethoxazole, [$^2H_6$]-trimethoprim, and [$^2H_6$]-venlafaxine were supplied by Sigma-Aldrich; [$^2H_5$]-acetaminophen, [$^2H_4$]-flecainide, and [$^2H_4$]-lorazepam by Alsachim (France). Individual standard solutions of targeted compounds were prepared at the concentration level of 2000 mg L^{-1} in MeOH and stored in amber glass vials at -20 °C in the dark. Working standard solutions were prepared by appropriate dilution of stock solutions in MeOH/water (10:90, v/v). High-purity water was obtained from a Milli-Q water purification Millipore system (USA).

For the quantification of metals, metalloids, and other major elements, individual ICP-MS standard solutions of 1000 mg L^{-1} of As, Ca, Cd, Cr, Cu, Fe, K, Na, Ni, Mg, Mn, P, Pb, and Zn were purchased from Fluka (St Louis, United States). An internal standard solution containing 10 mg L^{-1} of Li, Sc, Ge, Y, In, Tb, and Bi, and tuning solution containing 10 mg L^{-1} of Ce, Co, Li, Mg, Tl, and Y were obtained from Agilent Technologies (Santa Clara, United States). Certified quality control solutions were purchased from CPI International (Santa Rosa, United States). Multi-elemental standard stock solutions of 10 mg L^{-1} were prepared in ultrapure water from a Millipore Milli-Q water purification system (Darmstadt, Germany) containing 1% (v/v) of nitric acid by dilution of individual ICP-MS standards and used for preparing daily external calibration solutions.

Sample treatment for the extraction of pharmaceuticals and transformation products.

Freeze-dried soil samples were ground to obtain more homogeneous samples with an agate mortar. The sample extraction procedure was based on EPA Method 1694 with some modifications (USEPA, 2007). Briefly, 1 g of sample was weighed and joined with 200 µL of a solution of isotope-labelled internal standards of 40 µg L^{-1}. Then, the samples were placed in a fume hood overnight to allow the evaporation of methanol, achieving a concentration of 8 ng g^{-1} for each pharmaceutical.

For the extraction protocol under basic conditions (AAA, ACE, ATE, CBZ, CBZPOX, CIT, CLA, COD, COT, DAA, DIA, DIC, ENA, FAA, FLE, GEM, HDIC, IBU, LIN, MACE, METRO, NIC, OVEN, PRO, TRI, VAL, and VEN), a triplicate sequential extraction was carried out; we added 20 mL MeCN and sonicated the mixture in an ultrasonic bath for 30 min (Fisherbrand Model FB11201, Fisher Scientific, USA). Prior to the second extraction, 15 mL of ultrapure water at pH 10 was added to the soil and vortexed to resuspend soils. Supernatants were then separated by centrifugation (3000 rpm, 5 min) (centrifuge, 5810R Eppendorf ™, USA) and concentrated to a volume of 10 mL. The solution was diluted to 100 mL with ultrapure water (pH value adjusted to 10) and subjected to a solid-phase extraction (SPE) process using Oasis HLB cartridges (1 g, 20 mL, Waters, USA), previously conditioned with 20 mL of MeOH, 6 mL of ultrapure water, and 6 mL of basified ultrapure water adjusted to pH=10 with NH_4OH 32% (v/v). After the aqueous sample was loaded, the cartridge was washed with 20 mL of ultrapure water and dried under a vacuum for 5 min. Retained analytes were eluted with two aliquots of 10 mL of MeOH. The organic extract was evaporated to dryness using a Speed Vac concentrator (Thermo Scientific, USA) and reconstituted in 4 mL of MeOH:H_2O (10: 90, v/v). Finally, 1 mL aliquots were centrifuged for 5 min at 13,000 rpm and transferred to an amber glass vial prior to analysis.

For the extraction protocol under acidic conditions (ATEAC, LOR, N4ACE, and SUL), a triplicate sequential extraction was carried out by adding 20 mL MeCN and sonicating the mixture in an ultrasonic bath for 30 min (Fisherbrand Model FB11201, Fisher Scientific, USA). Prior to the second extraction, 15 mL of phosphate buffer at pH 2 was added to the soil and vortexed to resuspend the soils. Supernatants were then separated by centrifugation (3000 rpm, 5 min) (centrifuge, 5810R Eppendorf ™, USA) and concentrated to a volume of 10 mL. Then, 250 mg of $Na_2EDTA{\cdot}2H_2O$ was added and the extract was brought to a volume of 100 mL with ultrapure water. The solution was subjected to a solid-phase extraction (SPE) process using Oasis HLB cartridges (1 g, 20 mL, Waters, USA), previously conditioned with 20 mL of MeOH, 6 mL of ultrapure water, and 6 mL of acidified ultrapure water adjusted to pH = 2.0 ±0.5 with HCl. After the acid aqueous sample was loaded, the cartridge was washed with 20 mL of ultrapure water and dried under a vacuum for 5 min. Retained analytes were eluted with two aliquots of 10 mL of MeOH. The organic extract was evaporated to dryness and reconstituted in 4 mL of MeOH:H_2O (10:90, v/v). Finally, 1 mL aliquots were centrifuged for 5 min at 13,000 rpm and transferred to an amber glass vial prior to analysis.

Freeze-dried leaf samples (0.5 g) were weighed into a 50 mL PP centrifuge tube and 100 µL of a solution of isotope-labelled internal standards of 60 µg L^{-1} was added. Then, the samples were placed in a fume hood overnight, achieving a concentration of 12 ng g^{-1} for each pharmaceutical. For the extraction, 10 mL of MeOH was added and the tube was shaken (1 min) and sonicated (15 min) in an ultrasonic bath (Fisherbrand Model FB11201, Fisher Scientific, USA). The extract was centrifuged for 15 min at 4000 rpm and the supernatant was collected. A double extraction was performed by adding 15 mL of MeOH. Both supernatants were evaporated to 1 mL and reconstituted in 200 mL of ultrapure water containing 30 mg L^{-1} of EDTA and subjected to a solid-phase extraction process using the following protocol: Oasis HLB solid-phase extraction cartridge (1g, 20 mL, Waters, USA), previously conditioned with 20 mL of MeOH and 20 mL of ultrapure water. After loading the sample (200 mL), 20 mL of ultrapure water was added, and then, the cartridge was dried under a vacuum (5 bar) to eliminate residual water. Analytes were eluted with two aliquots of 6 mL of MeOH. The organic extract was evaporated to dryness at 45 °C, 0.2 Torr using a Speed Vac concentrator (Thermo Scientific, USA), and then reconstituted in 4 mL of MeOH: H_2O (10:90, v/v) and vortex stirring for 1 min. Reconstituted samples were centrifuged for 5 min at 13,000 rpm (MiniSpin centrifuge, Eppendorf, USA) and transferred to an amber glass vial prior to analysis.

Freeze-dried root samples (0.25 g) were weighed and mixed with 50 µL of a solution of isotope-labelled internal standards of 60 µg L^{-1}. Then, the samples were placed in a fume hood overnight, achieving a concentration of 12 ng g^{-1} for each pharmaceutical. A double extraction was performed by adding 7.5 mL of MeOH and performing sonication in an ultrasonic bath (Fisherbrand Model FB11201, Fisher Scientific, USA) for 15 min. The extracts were centrifuged, and the supernatants were mixed. For the analysis of root samples, two different aliquots (2.5 mL) were diluted to 50 mL with ultrapure water. One of them was adjusted to an 8–9 pH value for the quantification of CAR, CIT, CLA, GEM, IBU, LIN, LOR, MET, OVEN, PRO, TRI, and VEN. Then, diluted samples were subjected to a clean-up and preconcentration process by the application of the following solid-phase extraction protocol: Oasis HLB solid-phase extraction cartridge (200 mg, 6 mL, Waters, USA), previously conditioned with 6 mL of MeOH, 6 mL of ultrapure water, and 6 mL of ultrapure water at pH 8–9 (only for basic aliquot). After loading the sample, 10 mL of ultrapure water was added, and then, the cartridge was dried under a vacuum (5 bar) to eliminate residual water. Analytes were eluted with three aliquots of 4 mL of MeOH. The organic extract was evaporated to dryness at 45 °C, 0.2 Torr using a Speed Vac concentrator (Thermo Scientific, USA), and then reconstituted in 500 µL of MeOH: H_2O (10:90, v/v) and subjected to vortex stirring for 1 min. Reconstituted samples were centrifuged for 5 min at 13,000 rpm (MiniSpin centrifuge, Eppendorf, USA) and transferred to an amber glass vial prior to analysis.

Table S1. Instrumental parameters and chromatographic conditions for pharmaceutical and transformation product analysis by LC-MS/MS.

**Triple quadrupole (MS/MS) parameters**		
Ionization mode	Positive/negative	
Sheath gas temperature	350 °C	
Sheath gas flow	11 L min^{-1}	
Drying gas temperature	250 °C	
Drying gas flow	13 L min^{-1}	
Nebulizer press	25 psi	
Capillary voltage	4000 V (ESI+), 3000 V (ESI-)	
Nozzle voltage	500 V	
Δ EMV	400 V	
**Chromatographic conditions**		
Positive ionization mode	Chromatographic column	Kinetex Biphenyl, 50 x 3 mm x 2.7 µm (Phenomenex)
	Mobile phases	0.1 % (v/v) formic acid in ultrapure water (A) 0.1 % (v/v) formic acid in methanol (B)
	Elution mode	Initial mobile phase composition (2% B) constant for 1 min, followed by a linear gradient to 100 % B up to 30 min, and kept for 5 min at 100 % B
	Flow rate	0.6 mL min^{-1}
	Column temperature	40 °C
	Injection volume	20 µL
Negative ionization mode	Chromatographic column	Poroshell 120 EC-C18, 50 x 3 mm x 2.7 µm (Agilent Technologies)
	Mobile phases	1 mM ammonium fluoride in ultrapure water (A) Methanol/acetonitrile (65:35, v/v)
	Elution mode	Initial mobile phase composition (5 % B) constant for 1 min, followed by a linear gradient to 100 % B up to 12 min, and kept for 5 min at 100 % B
	Flow rate	0.6 mL min^{-1}
	Column temperature	40 °C
	Injection volume	20 µL

Table S2. Retention times (t_R), collision energies (CE), precursors and product ions (Q; quantifier, and q; qualifier) selected for the analysis of pharmaceuticals and transformation products (in italics) in Multiple Reaction Mode (MRM).

**Compound**	**Formula**	**t_R (min)**	**Molecular ion**	**Precursor (m/z)**	**Product ion (m/z)**	**CE (V)**	**MRM transition**	**Abundance (q/Qx 100) (%)**
3-Metoxy-acetaminophen	$C_9H_{11}NO_3$	4.07	$[M+H]^+$	182	108	14	Q	
					80.1	40	q_1	44
					140	18	q_2	14
4-Acetamidoantipyrine	$C_{13}H_{15}N_3O_2$	10.9	$[M+H]^+$	246.0	228.0	12	Q	
					204.1	12	q_1	36
4-Formylaminoantipyrine	$C_{12}H_{13}N_3O_2$	10.9	$[M+H]^+$	232.2	214.0	12	Q	
					203.9	12	q_1	52
4-Hydroxy-diclofenac	$C_{14}H_{11}Cl_2NO_3$	19.4	$[M+H]^+$	311.8	229.9	36	Q	
					265.9	18	q_1	88
Acetaminophen	$C_{10}H_9NO_2$	2.5	$[M+H]^+$	152.0	109.9	20	Q	
					93.0	24	q_1	28
Atenololic acid	$C_{13}H_{19}NO_4$	8.2	$[M+H]^+$	267.8	145.1	30	Q	
					191.0	14	q_1	60
Atenolol	$C_{14}H_{22}N_2O_3$	5.9	$[M+H]^+$	266.8	189.9	19	Q	
					145.1	31	q_1	133
Carbamazepine	$C_{15}H_{12}N_2O$	18.2	$[M+H]^+$	237.0	193.8	21	Q	
					192.8	40	q_1	22

Carbamazepine epoxide	$C_{15}H_{12}N_2O_2$	16.2	$[M+H]^+$	253.0	180.0	36	Q	
					235.8	12	q_1	56
Citalopram	$C_{20}H_{21}FN_2O$	16.5	$[M+H]^+$	325.0	108.9	20	Q	
					261.9	19	q_1	89
Clarithromycin	$C_{38}H_{69}NO_{13}$	19.9	$[M+H]^+$	748.4	590.4	19	Q	
					157.9	38	q_1	16
Codeine	$C_{18}H_{21}NO_3$	8.9	$[M+H]^+$	300.0	215.0	32	Q	
					225.0	27	q_1	65
Cotinine	$C_{10}H_{12}N_2O$	3.27	$[M+H]^+$	177.1	80.1	24	Q	
					98	25	q_1	26
Diazepam	$C_{16}H_{13}ClN_2O$	22.9	$[M+H]^+$	285	153.9	32	Q	
					192.9	32	q_1	100
					256.9	22	q_2	72
Diclofenac	$C_{14}H_{11}Cl_2NO_2$	22.9	$[M+H]^+$	295.8	214.7	20	Q	
					249.9	12	q_1	72
Enalapril	$C_{20}H_{28}N_2O_5$	16.5	$[M+H]^+$	377.2	234	22	Q	
					303	16	q_1	27
Flecainide	$C_{17}H_{20}F_6N_2O_3$	15.6	$[M+H]^+$	415.1	398.0	24	Q	
					300.9	40	q_1	50
Gemfibrocil	$C_{15}H_{22}O_3$	11.9	$[M-H]^-$	249.0	121.0	10	Q	
					127.0	8	q_1	7
Ibuprofen	$C_{13}H_{18}O_2$	10.8	$[M-H]^-$	205.1	161.1	4	Q	
Lincomycin	$C_{18}H_{34}N_2O_6S$	7.65	$[M+H]^+$	407.2	126.1	32	Q	
					359	18	q_1	6
Lorazepam	$C_{15}H_{10}ClN_2O$	19.4	$[M+H]^+$	321.1	274.8	24	Q	
					228.9	36	q_1	33
					302.9	29	q_2	4
Metronidazole	$C_6H_9N_3O_3$	4.2	$[M+H]^+$	172.0	128.0	10	Q	
					82.0	30	q_1	47
N4-acetylsulfamethoxazole	$C_{12}H_{13}N_3O_4S$	13.4	$[M+H]^+$	296	133.9	26	Q	
					108	34	q_1	54
Nicotine	$C_{10}H_{14}N_2$	1.8	$[M+H]^+$	163.1	130.0	30	Q	
					131.9	16	q_1	100
o-Desmethylvenlafaxine	$C_{16}H_{25}NO_2$	10.1	$[M+H]^+$	263.8	58.2	25	Q	
					246	10	q_1	40
Propranolol	$C_{16}H_{22}ClNO_2$	15.4	$[M+H]^+$	259.7	115.9	20	Q	
					155	24	q_1	41
					182.9	20	q_2	78
Sulfamethoxazole	$C_{10}H_{11}N_3O_3S$	11.7	$[M+H]^+$	253.9	92.0	31	Q	
					155.8	14	q_1	88
Trimethoprim	$C_{14}H_{18}N_4O_3$	8.9	$[M+H]^+$	291.2	230	25	Q	
					123	24	q_1	56
Valsartan	$C_{24}H_{29}N_5O_3$	22.6	$[M+H]^+$	436.0	235.0	20	Q	
					291.0	18	q_1	87
Venlafaxine	$C_{17}H_{27}NO_2$	14.3	$[M+H]^+$	278.2	58.0	18	Q	
					260.0	8	q_1	52

Table S3. Instrumental detection (LODi) and quantification (LOQi) limits for pharmaceuticals and transformation products (in italics). Absolute and relative recoveries (%) and relative standard deviation values (RSD, %) (n = 3) for pharmaceuticals and transformation products (in italics) obtained in soil and plant samples (roots and leaves).

**Compound**	**LODi, LOQi (ng L^{-1})**	**Soil**		**Roots**		**Leaves**	
		**R (%); RSD (%)**	**MQL (ng g^{-1})**	**R (%); RSD (%)**	**MQL (ng g^{-1})**	**R (%); RSD (%)**	**MQL (ng g^{-1})**
3-Metoxy-acetaminophen (*)	3, 10	No data		75; 4	0.16	72; 1(L_{int})<br>44; 16(L_{ext})	0.06<br>0.20
4-Acetamidoantipyrine (4-AAA)	3, 10	78; 4	0.05	81; 5	0.18	62; 20	0.21
4-Dimethilaminoantipyrine (4-DAA)	3, 10	<10; 5	1.00	73; 12	0.18	50; 20	0.15
4-Formylaminoantipyrine (4-FAA)	6, 20	91; 7	0.10	73; 2	0.31	80; 12	0.20
4-Hidroxi-diclofenaco (*)	3, 10	<10; 20	2.00	<10; 9	8.50	26; 3(L_{int})<br>10; 16(L_{ext})	1.00<br>2.40
Acetaminophen	20, 50	105; 22[a]	2.50	117; 1[a]	0.85	50; 17	1.10
Atenololic acid	10, 30	74; 2	0.20	29; 32	1.38	58; 18	0.40
Atenolol	3, 10	85; 11[a]	0.05	87; 3[a]	0.18	82; 10[a]	0.20
Carbamazepine	3, 10	102; 12[a]	0.04	88; 2[a]	0.12	86; 20[a]	0.16
Carbamazepine epoxide (*)	3, 10	70; 9	0.06	73; 9	1.50	84; 9(L_{int})<br>45; 16(L_{ext})	0.10<br>0.20
Citalopram	3, 10	97; 9[a]	0.20	88; 28[a]	0.50	74; 8	0.20
Clarithromycin	3, 10	77; 17[a]	0.05	86; 6[a]	0.26	89; 15[a]	0.10
Codeine	3, 10	88; 8[a]	0.05	111; 1[a]	0.21	97; 9[a]	0.15
Cotinine	3, 10	85; 8	0.05	86; 3	0.60	55; 18	0.20
Diazepam (*)	3, 10	52; 10	0.10	57; 5	0.21	51; 20(L_{int})<br>34; 4(L_{ext})	0.20<br>0.30
Diclofenac (*)	50, 100	45; 16	1.00	38; 2	3.50	39; 4(L_{int})<br>21; 10(L_{ext})	2.30<br>4.15
Enalapril	30, 100	81; 2	0.50	85; 7	0.77	70; 8	1.30
Flecainide	30, 100	79; 2[a]	0.80	27; 9	15.8	70; 15	1.40
Gemfibrozil	3, 10	91; 9	0.50	64; 9	1.73	54; 20	1.80
Ibuprofen	30, 100	98; 7[a]	0.43	93; 2[a]	1.20	89; 15[a]	0.90
Lincomycin (*)	3, 10	72; 4	0.06	83; 10	0.14	65; 1(L_{int})<br>33; 17(L_{ext})	0.15<br>0.30
Lorazepam	6, 20	69; 14	0.12	46; 2	0.50	95; 2	0.40
Metronidazole	3, 10	76; 2	0.05	71; 1	0.16	45; 11	0.30
N4-acetylsulfamethoxazole (*)	6, 20	67; 5	0.12	34; 3	1.77	52; 9(L_{int})<br>24; 15(L_{ext})	0.40<br>0.70
Nicotine	20. 50	71; 13	0.30	93; 7[a]	0.60	93; 12[a]	4.20
o-Desmethylvenlafaxine	10, 30	53; 10	0.40	65; 8	0.20	30; 12	0.90
Propranolol	10, 30	73; 16[a]	0.30	86; 26[a]	0.52	92; 4[a]	0.50
Sulfamethoxazole	3, 10	106; 6[a]	0.04	117; 5[a]	2.00	98; 8[a]	0.60
Trimethoprim	3, 10	76; 8[a]	0.07	88; 11[a]	0.75	107; 14[a]	0.20
Valsartan	30, 100	74; 8	0.60	69; 6	1.71	64; 16	1.50
Venlafaxine	30, 100	116; 14[a]	1.00	109; 4[a]	2.50	109;7[a]	1.10

[a] Relative recoveries (%) based on the peak area of reference standard with the correction of internal standard area.

[*] Compounds with RSD > 20% in recoveries achieved in leaf samples. Application of different methodological recoveries for determining the concentrations in external (Lext) and internal leaves (Lint).

Table S4. ICP-MS instrumental parameters.

**ICP-MS instrumental parameters**	
Forward power	1550 W
Plasma gas flow rate	15 L min^{-1}
Carrier gas flow rate	1.10 L min^{-1}
Sample/skimmer cones	Ni
Collision gas	Helium
Collision gas flow rate	4.3 mL min^{-1}
Isotope monitored	^{23}Na, ^{24}Mg, ^{25}Mg, ^{31}P, ^{39}K, ^{43}Ca, ^{44}Ca, ^{52}Cr, ^{53}Cr, ^{54}Fe, ^{56}Fe, ^{55}Mn, ^{58}Ni, ^{60}Ni, ^{63}Cu, ^{64}Zn, ^{65}Cu, ^{66}Zn, ^{75}As, ^{111}Cd, ^{114}Cd, ^{206}Pb, ^{207}Pb, ^{208}Pb
Internal standard	^{6}Li, ^{45}Sc, ^{72}Ge, ^{89}Y, ^{115}In
Dwell time	100 ms
Replicates	3

Table S5. Instrumental detection and quantification limits (LODi and LOQi) for element analysis in water samples by ICP-MS. Methodological quantification limits (MQL) for elements analysis in soil and plant samples by ICP-MS prior to acid digestion.

**Element**	**LODi µg L^{-1}**	**LOQi µg L^{-1}**	**MQL mg kg^{-1}**
As	0.03	0.05	0.1
Ca	20	50	100
Cd	0.003	0.01	0.02
Cr	0.02	0.05	0.1
Cu	0.1	0.3	0.6
Fe	0.3	1.0	2.0
K	3.0	10	20
Mg	0.3	1.0	2.0
Mn	0.1	0.3	0.6
Na	2.0	5.0	10
Ni	0.1	0.3	0.6
P	5.0	14	30
Pb	0.01	0.03	0.06
Zn	0.3	1.0	2.0

Table S6. Average concentrations of pharmaceutical and transformation products in the interstitial water from CA and TM conditions over the 45-day irrigation period.

	**Interstitial water concentration ng L^{-1}**											
	**CA**						**TM**					
**Pharmaceuticals**	**Week 1**	**Week 2**	**Week 3**	**Week 4**	**Week 5**	**Week 6**	**Week 1**	**Week 2**	**Week 3**	**Week 4**	**Week 5**	**Week 6**
**Carbamazepine**	106.9	104.7	95.8	84.4	32.9	85.3	128.5	114.5	103.2	100.0	32.1	223.2
**Carbamazepine epoxide**	29.7	28.9	28.5	31.8	21.6	23.1	36.4	30.8	32.9	34.5	22.9	34.1
**Sulfamethoxazole**	45.1	46.0	ND	ND	28.1	ND	44.8	45.3	27.6	28.0	38.1	87.6
**Acetaminophen**	31.1	23.8	ND	ND	ND	ND	47.4	35.7	ND	ND	ND	ND
**4-Formylamino antipyrine**	16.8	16.8	ND	ND	ND	ND	15.3	<LOQ	ND	ND	ND	ND
**N4-acetylsulfamethoxazole**	ND	ND	ND	ND	ND	ND	ND	33.5	ND	ND	ND	ND
**Diazepam**	<LOQ	<LOQ	<LOQ	<LOQ	<LOQ	<LOQ	<LOQ	<LOQ	<LOQ	<LOQ	<LOQ	17.5
**Lincomycin**	ND	ND	ND	ND	ND	ND	ND	ND	ND	ND	ND	9.7
**Lorazepam**	ND	ND	ND	ND	ND	ND	ND	ND	ND	ND	ND	38.3
**Atenololic acid**	ND	ND	ND	ND	ND	ND	ND	ND	ND	ND	ND	47.1

Table S7. Total average soil contents of pharmaceuticals and transformation products (dry weight) from CA and TM conditions after the 45-day irrigation period.

Soil concentration ng g^{-1}		
**Pharmaceuticals**	**CA**	**TM**
Flecainide	12.32	12.85
Carbamazepine	5.85	6.31
Atenololic acid	2.32	2.58
Clarithromycin	1.13	0.97
Citalopram	0.94	0.83
Ibuprofen	0.49	ND
Lorazepam	0.46	ND
Diazepam	0.38	0.43
Carbamazepine epoxide	0.20	0.31
Cotinine	0.07	<LOQ
Atenolol	<LOQ	0.08

Table S8. Average concentrations of pharmaceuticals and transformation products in external leaves, internal leaves, and roots of lettuce plants grown in CA and TM conditions after the 45-day irrigation period.

Pharmaceuticals	CA			TM		
	**External leaves** ng g-1	**Internal leaves** ng g-1	**Roots** ng g-1	**External leaves** ng g-1	**Internal leaves** ng g-1	**Roots** ng g-1
**Carbamazepine**	30.31	4.31	1.11	22.50	3.93	1.26
**Carbamazepine epoxide**	4.14	1.02	0.18	3.41	0.47	0.18
**Venlafaxine**	5.11	0.95	2.64	7.75	0.75	1.51
**Diazepam**	0.50	<LOQ	0.32	0.32	<LOQ	0.40
**Flecainide**	3.67	<LOQ	<LOQ	6.75	<LOQ	<LOQ
**Ibuprofen**	<LOQ	ND	2.52	ND	ND	0.87
**Nicotine**	<LOQ	<LOQ	0.68	<LOQ	<LOQ	1.76
**Citalopram**	0.86	ND	<LOQ	ND	ND	<LOQ

Table S9. Pearson's correlation coefficients calculated among pharmaceutical and transformation products' concentrations in environmental matrices (lettuce, interstitial water, and soil) and compound physico-chemical parameters in Ph condition.

	Plant	*Soil*	*Int. water*	*Molecular mass*	*log Kow*	*log Dow (pH 8.7)*	*Neutral fraction*	*Ionic fraction*	*Charge state*	*HDB*	*HBA*
Plant	1.00										
Soil	**0.82****	1.00									
Int. water	**0.46***	0.32	1.00								
Molecular mass	-0.02	0.26	-0.06	1.00							
log Kow	0.15	0.20	-0.01	0.24	1.00						
log Dow (pH 8.7)	**0.59****	**0.66****	0.10	0.23	**0.59****	1.00					
Neutral fraction	0.14	0.00	0.08	-0.31	-0.34	0.22	1.00				
Ionic fraction	-0.14	0.00	-0.08	0.31	0.34	-0.22	**-1.00****	1.00			
Charge state	0.06	0.22	-0.08	**0.42***	0.32	-0.02	**-0.74****	**0.74****	1.00		
HDB	-0.17	-0.06	0.05	**0.58****	-0.03	-0.19	-0.26	0.26	0.32	1.00	
HBA	-0.29	-0.04	-0.13	**0.85****	-0.05	-0.08	-0.19	0.19	0.32	**0.68****	1.00

* = $p < 0.05$ a ** = $p < 0.01$, respectively (n = 30). HDB = hydrogen-bond donors and HBA = hydrogen-bond acceptors.

Table S10. Pearson's correlation coefficients calculated among pharmaceutical and transformation products' concentrations in environmental matrices (lettuce, interstitial water, and soil) and compound physico-chemical parameters in Ph-TM condition.

	Plant	*Soil*	*Int. water*	*Molecular mass*	*log Kow*	*log Dow (pH 8.7)*	*Neutral fraction*	*Ionic fraction*	*Charge state*	*HDB*	*HBA*
Plant	1.00										
Soil	**0.81****	1.00									
Int. water	**0.44***	0.22	1.00								
Molecular mass	-0.02	0.26	-0.05	1.00							
log Kow	0.15	0.21	0.03	0.24	1.00						
log Dow (pH 8.7)	**0.61****	**0.68****	0.10	0.23	0.59	1.00					
Neutral fraction	0.13	-0.03	0.08	-0.31	-0.34	0.22	1.00				
Ionic fraction	-0.13	0.03	-0.08	0.31	0.34	-0.22	**-1.00****	1.00			
Charge state	0.07	0.27	-0.08	**0.42***	0.32	-0.02	**-0.74****	**0.74****	1.00		
HDB	-0.18	-0.07	0.00	**0.58****	-0.03	-0.19	-0.26	0.26	0.32	1.00	
HBA	-0.29	-0.02	-0.14	0.85	-0.05	-0.08	-0.19	0.19	0.32	**0.68****	1.00

* = $p < 0.05$ and ** = $p < 0.01$, respectively (n = 30). HDB = hydrogen-bond donors and HBA = hydrogen-bond acceptors.
